# Influenza A virus infection disrupts oligodendrocyte homeostasis and alters the myelin lipidome in the adult mouse

**DOI:** 10.1186/s12974-023-02862-2

**Published:** 2023-08-19

**Authors:** Allison Y. Louie, Justin S. Kim, Jenny Drnevich, Payam Dibaeinia, Hisami Koito, Saurabh Sinha, Daniel B. McKim, Katiria Soto-Diaz, Romana A. Nowak, Aditi Das, Andrew J. Steelman

**Affiliations:** 1 Neuroscience Program, 2325/21 Beckman Institute, 405 North Mathews Ave., Urbana, IL 61801 USA; 2 Division of Nutritional Sciences, University of Illinois at Urbana-Champaign, 1201 W. Gregory Dr., Urbana, IL 61801 USA; 3 School of Chemistry and Biochemistry, Georgia Institute of Technology, 3306, IBB, Parker H. Petit Institute for Bioengineering and Biosciences, 315 Fernst Dr. NW, Atlanta, GA 30332 USA; 4 Department of Comparative Biosciences, University of Illinois at Urbana-Champaign, 3516 Veterinary Medicine Basic Sciences Bldg., 2001 South Lincoln Avenue, Urbana, IL 61802 USA; 5 Roy J. Carver Biotechnology Center, University of Illinois at Urbana-Champaign, Urbana, IL USA; 6 Department of Computer Science, University of Illinois at Urbana-Champaign, 201 North Goodwin Avenue, Urbana, IL 61801 USA; 7 Department of Pharmaceutical Sciences, Josai University, 1-1 Keyakidai, Sakado-shi, Saitama, 350-0295 Japan; 8 Carl R. Woese Institute for Genomic Biology, University of Illinois at Urbana-Champaign, 1206 West Gregory Dr., Urbana, IL 61801 USA; 9 Department of Bioengineering, Cancer Center at Illinois, Beckman Institute for Advanced Science and Technology, University of Illinois at Urbana-Champaign, 405 N. Mathews Ave., Urbana, IL 61801 USA; 10 Department of Animal Sciences, University of Illinois at Urbana-Champaign, 1201 W. Gregory Dr., Urbana, IL 61801 USA; 11 The Wallace H. Coulter Department of Biomedical Engineering, Georgia Institute of Technology, Atlanta, USA

**Keywords:** Myelin, Myelin plasticity, Lipid, Lipidome, Microglia, Oligodendrocyte, Influenza virus, Inflammation, Systemic inflammation

## Abstract

**Background:**

Recent data suggest that myelin may be altered by physiological events occurring outside of the central nervous system, which may cause changes to cognition and behavior. Similarly, peripheral infection by non-neurotropic viruses is also known to evoke changes to cognition and behavior.

**Methods:**

Mice were inoculated with saline or influenza A virus. Bulk RNA-seq, lipidomics, RT-qPCR, flow cytometry, immunostaining, and western blots were used to determine the effect of infection on OL viability, protein expression and changes to the lipidome. To determine if microglia mediated infection-induced changes to OL homeostasis, mice were treated with GW2580, an inhibitor of microglia activation. Additionally, conditioned medium experiments using primary glial cell cultures were also used to test whether secreted factors from microglia could suppress OL gene expression.

**Results:**

Transcriptomic and RT-qPCR analyses revealed temporal downregulation of OL-specific transcripts with concurrent upregulation of markers characteristic of cellular stress. OLs isolated from infected mice had reduced cellular expression of myelin proteins compared with those from saline-inoculated controls. In contrast, the expression of these proteins within myelin was not different between groups. Similarly, histological and immunoblotting analysis performed on various brain regions indicated that infection did not alter OL viability, but increased expression of a cellular stress marker. Shot-gun lipidomic analysis revealed that infection altered the lipid profile within the prefrontal cortex as well as in purified brain myelin and that these changes persisted after recovery from infection. Treatment with GW2580 during infection suppressed the expression of genes associated with glial activation and partially restored OL-specific transcripts to baseline levels. Finally, conditioned medium from activated microglia reduced OL-gene expression in primary OLs without altering their viability.

**Conclusions:**

These findings show that peripheral respiratory viral infection with IAV is capable of altering OL homeostasis and indicate that microglia activation is likely involved in the process.

**Supplementary Information:**

The online version contains supplementary material available at 10.1186/s12974-023-02862-2.

## Background

Myelin, the fatty, insulating substance that constitutes brain white matter, forms compact concentric sheaths around axons and increases neuronal conduction velocity by 10- to 100-fold at a fraction of the energy demand [1, 2]. In the central nervous system (CNS), oligodendrocytes (OLs) are responsible for internodal myelin segment formation, which is required for proper motor function and is imperative for higher order cognition, such as reading, vocabulary and executive decision-making [3, 4]. In contrast, hypomyelination and demyelination are pathological features of a myriad of neurological diseases, with white matter abnormalities being a feature of schizophrenia [5–7], cognitive decline due to aging [8], Alzheimer’s disease [9], multiple sclerosis [10] and depression [11].

It has been known for some time that alterations to OL homeostasis affect cognition and behavior, but the notion that myelin remains static during adulthood has only recently been challenged. For instance, even though the turnover of brain OLs is slow, myelin itself has been shown to be continuously exchanged [12]. Additional evidence implicating myelination as an ongoing and dynamic process is illustrated by the findings that changes to white matter structures occur when learning complex motor skills such as playing an instrument [13] or juggling [14]. Myelin is also affected by other environmental factors such as diet, exercise, and the microbiome [15].

The structural integrity of myelin, attributed to proteins such as myelin basic protein (MBP) [16], proteolipid protein (PLP) [17], and apolipoproteins [18], as well as the interaction between lipids located within the myelin sheath, is essential for its proper functionality [19]. Indeed, alterations to the myelin lipid profile alone can result in ultrastructural changes to myelin [20], and deletion of particular lipid species within myelin can have functional consequences on conduction velocity despite overall normal-appearing structure [21, 22]. As such, lipidomics has emerged as a powerful tool with which to elucidate changes to the lipid species of myelin [23]. However, the influence of environmental factors on the myelin lipidome has not yet been elucidated. Since both the structure and biochemical profile of myelin affect conduction velocity, alterations to myelin thickness, length, or lipid composition might affect neurocircuitry, modulate synaptic plasticity, and act to influence behavior.

Systemic inflammation induces behavioral modifications that mimic those following perturbations to myelin [24]. For instance, patients with severe respiratory infections caused by influenza or SARS viruses can develop encephalopathy characterized by confusion, depressed mood, anxiety, impaired memory, insomnia, mania and psychosis even in the absence of CNS invasion [25, 26]. In survivors, these manifestations may be long lasting [26, 27]. While the underlying factors dictating these behavior changes are unknown, they are thought to involve high levels of systemic inflammation. It is known that microglia become reactive in response to systemic inflammation [28] and/or viral infection [29–31] and that this phenomenon is correlated with altered behavior. Microglial reactivity is also a prominent feature of most white matter diseases and the proinflammatory cytokines they produce during systemic inflammation influence OL homeostasis [32]. Whether aberrant inflammation brought on by viral infection alters myelin plasticity in adults has not yet been investigated. Since both proteins and lipids contribute to the structure and thus function of myelin, in the current study, we comprehensively examine how systemic inflammation, induced by infection with a respiratory pathogen, influences the transcriptional, translational, and biochemical profile of myelin. Here, we report that respiratory infection with influenza A virus (IAV) suppresses OL-specific transcription, increases a disease-specific marker of OL stress, and alters the lipid profile of myelin in the adult mouse CNS without altering cell viability or causing demyelination.

## Materials and methods

### Animals and viral infection

Male and female C57BL/6J mice were obtained from Jackson Laboratories (#000664). Unless otherwise indicated, males were used to generate experimental data. Animals were housed 4–5 per cage under constant 12 h light/dark cycles (10 am-10 pm) and constant temperature and fed a standard rodent diet (Envigo Diet No. 2918) ad libitum. Mice (aged 8–11 weeks) were anesthetized with 3% isoflurane and then inoculated with either sterile phosphate-buffered saline (PBS) or one hemagglutination unit (HAU) of mouse-adapted human influenza A virus (strain A/Puerto Rico/8/1934 H1N1) diluted in sterile PBS. The total inoculation volume was 30 µl. Animals were weighed daily and then euthanized by CO_2_ asphyxiation at days 8 and 16 p.i. for follow-up experiments. Weight data were pooled from all experiments. Sprague Dawley rats (Charles River Laboratories, Inc.; #400) were used to generate primary glial cultures for conditioned medium experiments. All animal care protocols were in accordance with National Institutes of Health Guidelines for Care and Use of Laboratory Animals and were approved by the University of Illinois Laboratory Animal Care and Use Committee.

### Bulk RNA-sequencing re-analysis

In the current study, we provide an extensive analysis of downregulated genes in the cerebellum and spinal cord of influenza inoculated mice using a dataset that we have previously published [33]. The RNA-sequencing datasets associated with this paper have been deposited into the Gene Expression Omnibus database (https://www.ncbi.nlm.nih.gov/geo/) and can be accessed using the accession no. GSE96870. Gene ontology and pathway analyses were performed on downregulated genes that achieved an FDR < 0.05 using DAVID Bioinformatics Resources 6.8 (https://david-d.ncifcrf.gov/).

### Tissue extraction and gene expression by RT-qPCR

Mice were euthanized at days 8 and 16 p.i. and intracardially perfused with 30 ml of sterile PBS. Brains were harvested, bisected, and immediately placed in RNALater (Thermo Scientific #AM7020) at 4 °C for 24–48 h and then stored at − 80 °C for later use. The medial prefrontal cortex (mPFC), cerebellum, and hippocampus were hand-dissected with the aid of a dissection microscope (Leica). The RNA from each brain region was isolated using TRIzol Reagent (Thermo Scientific #15596018) according to manufacturer’s instructions and purified using GeneJET RNA purification kit (Thermo Scientific #K0731). cDNA was obtained using the Reverse Transcription System (Promega #A3500) according to manufacturer’s instructions in a C1000 Touch Thermal Cycler (Bio-Rad Laboratories). Relative gene expression of select downregulated genes as indicated by RNA-sequencing results was determined using TaqMan Gene Expression Probe Assays (Integrated DNA Technologies, Table 1) on a QuantStudio 7 real time PCR system (Applied Biosystems, Thermo Scientific). All samples were run in duplicate. Expression levels were calculated as the average of two replicates for each biological sample from both groups (*n* = 3–10 animals per group) relative to *Actb* expression. Fold change was calculated using the formula 2^−∆∆Ct^.

**Table 1:** List of RT-qPCR primer and probe sequences

Target	Primer sequence	Probe sequence
Mouse		
*Actb*	F: 5'-GATT ACTGCTCTGGCTCCTAG-3' R: 5'-GACTCATCGTACTCCTGCTTG-3'	5'-/56-FAM/CTGGCCTCA/ZEN/CTGTCCACCTTCC/31ABkFQ/-3'
*Cnp*	F: 5'-AATTCTGTGACTACGGGAAGG-3' R: 5'-AGAGAGCAGAGATGGACAGT-3'	5'-/56-FAM/AGCAGGAGG/ZEN/TGGTGAAGAGATCGT A/31ABkFQ/-3'
*Cyp51*	F: 5'-GACTTT AATCCTGACCGCTACT-3' R: 5'-TCTCCAACACAACGATGACG-3'	5'-/ 56-FAM/TGGCACA T A/Z EN/GGCAAACTTCTCTCCTG /31ABkFQ/-3'
*Dhcr7*	F: 5'-CTCATT AACCTGTCCTTCGCT-3' R: 5'-GCAGATGTCGATGGTCTTCAG-3'	5'-/56-FAM/AACACGT AG/ZEN/ ATGGCCTGCAAGACA/31ABkFQ/-3'
*Hmgcs1*	F: 5'-CTGCT ATTCTGTCT ACCGCAA-3' R: 5'-TGAGTGAAAGATCATGAAGCCA-3'	5'-/56-FAM/TCCTTTCCC/ZEN/TCTTTCTGCCACTGG/31ABkFQ/-3'
*Idi1*	F: 5'-ATTGGTGTGAAGCGAGCA-3' R: 5'-CATCAGATTGGGCCTTGTAGT-3'	5'-/56-FAM/ AAGCCGAGT/ZEN/TGGGAAT ACCCTTGG/31ABkFQ/-3'
*Mag*	F: 5'-AGAGAGCAGAGATGGACAGT-3' R: 5'-CACCATACAACTGACCTCCAC-3'	5'-/56-FAM/CA TCGTCAA/Z EN/CACCCCCAACA TTGTG /31ABkFQ/-3'
*Mbp*	F: 5'-ACCCAAGATGAAAACCCAGTAG-3' R: 5'-CCTCCGTAGCCAAATCCTG-3'	5'-/56-FAM/ AGAA CA TTG/Z EN/TGACACCTCGAACACCA/31ABkFQ/-3'
*Mog*	F: 5'-CACTTGTGCCTACGATCCTC-3' R: 5'-AGTCCGATGGAGATTCTCTACT-3'	5'-/56-FAM/CACGAAGTT/Z EN/TTCCTCTCAGTCTGTGCT /31ABkFQ/-3'
*Msmo1*	F: 5'-CACAGACTCCTTCACCACAA-3' R: 5'-ATGTGCGT ATTCTGCTTCGAT-3'	5'-/56-FAM/TCCATCACG/ZEN/AGTTTCAGGCTCCATTT /31ABkFQ/-3'
*Nsdhl*	F: 5'-GTGTTCAGCCAGACTTCTCT-3' R: 5'-TGGTTTCGTTTGCAATCAACTC-3'	5'-/56-FAM/ ACTCAAATG/ZEN/ATCGACCTCAGCTTGCA/31ABkFQ/-3'
*Plin4*	F: 5'-GACTT ACAAACAGCAACAGACC-3' R: 5'-AAACTTCCCATGTCCTTGTCT-3'	5'-/56-FAM/TTCAGAAGG/ZEN/TTGGAGCAGCCCT/31ABkFQ/-3'
*Plp1*	F: 5'-GTTCCAAATGACCTTCCACCT-3' R: 5'-ATGAGTTTAAGGACGGCGAAG-3'	5'-/ 56-FAM/CACACT AGT /Z EN/TTCCCTGCTCACCTTCA/31ABkFQ/-3'
*Ugt8a*	F: 5'-CAAGACCAACGCTGCCT AA-3' R: 5'-CATGTTCCTGAGCACCACTT-3'	5'-/56-FAM/ AGCCCACTG/ZEN/CCAGAAGATCTGC/31ABkFQ/-3'
**Rat**		
*Actb*	F: 5’-GGCATAGAGGTCTTTACGGATG-3’ R: 5’-TCACTATCGGCAATGAGCG-3’	5’-/56-FAM/TCC TGG GTA /ZEN/TGG AAT CCT GTG GC/3IABkFQ/-3’
*Ugt8*	F: 5’-TCCTGAGCACCATCTACC C-3’ R: 5’-TGTTATGTACTGACGTAGCACTG-3’	5’-/56-FAM/AGC CCA CTG /ZEN/CCA GAA GAT CTG C/3IABkFQ/-3’

### Immunoblotting

Mice were euthanized at day 8 p.i. and intracardially perfused with 30 ml of sterile PBS. Brains were harvested, then mPFC, cerebellum, and hippocampus regions micro-dissected and sonicated in radioimmunoprecipitation assay (RIPA) buffer (Cell Signaling Technology, CST #9806) during two rounds of fifteen 1-s pulses. Samples were centrifuged at 14,000 × *g* for 10 min and supernatants collected into new tubes. Protein concentration was determined using DC™ Protein Assay Kit II (Bio-Rad #5000112) and samples boiled for 5 min with 2 × Laemmli Sample Buffer (Bio-Rad #1610737). SDS-PAGE was performed on samples along with protein standards ladder (Bio-Rad #1610374) using 4–20% Mini-PROTEAN® TGX™ Precast Protein Gels (Bio-Rad #4561096) with a Mini-PROTEAN® Tetra Vertical Electrophoresis Cell (Bio-Rad #165-8004) and transfer to Immobilon®-P PVDF Membrane (Millipore IPVH00010) was performed using a Mini Trans-Blot® Module (Bio-Rad #170-3935). Membranes were incubated with 5% non-fat dried milk (NFDM) in Tris-buffered saline containing 0.1% Tween-20 (TBST) for 1 h at room temperature and subsequently with primary antibody diluted at 1:1000 in 5% NFDM in TBST overnight at 4 °C with gentle rocking. The following primary antibodies were used: MAG (CST #9043), MOG (CST #96-457), SOX10 (Abcam #AB227680), PLIN4 (NovusBio #NBP2-13776), GAPDH (CST #5174). Membranes were washed in TBST for three rounds of 5 min each and then incubated with HRP-linked Anti-rabbit IgG (CST #7074) for 1 h at room temperature before additional washing in TBST. 1 × solution of SignalFire™ ECL Reagent (CST #6883) was prepared and applied to membranes, which were then imaged by chemiluminescence with increasing exposure of 10 s intervals with an ImageQuant LAS-4000 Luminescent Image Analyzer (FujiFilm).

### Immunohistochemistry

Mice were euthanized at day 8 p.i. and intracardially perfused with 30 ml of sterile PBS. Brains were harvested, bisected, and immediately submerged in cold 4% paraformaldehyde diluted in PBS. Tissues were post-fixed at 4 °C for 24 h and then cryoprotected in 30% sucrose diluted in PBS for 48 h at 4 °C. Brain halves were rapidly frozen in finely crushed dry ice, embedded in Tissue-Tek® O.C.T. Compound (Sakura #4583) in a dry ice-ethanol slurry, and then sectioned on a cryostat microtome (Leica CM 1950). Sagittal sections of 20 µm thickness were stored at − 80 °C for later use. Tissues were rehydrated in PBS for 20 min and then blocked with PBS containing 5% goat serum and 0.3% Triton-X 100 (PBST) for 1 h at room temperature. Tissue sections were then incubated with primary antibodies: anti-APC clone CC1 (1:500; Calbiochem #OP80), anti-SOX10 (1:200; Abcam #AB227680), anti-GFAP (1:1000; EMD Millipore #AB5541), and anti-Iba1 (1:1000; FUJIFILM Wako #019-19741) at 4 °C overnight. After washing with PBST for three rounds of 5 min each, tissues were incubated with fluorophore-conjugated secondary antibody at 1:1000 dilution for 1 h at room temperature before washing again with PBST. The following secondary antibodies were used: goat anti-mouse Alexa Fluor™ 594 (Thermo #A11005), goat anti-rabbit Alexa Fluor™ 488 (Thermo #A11008), and goat anti-chicken Alexa Fluor™ 594 (Thermo #A11041). Tissues were counter-stained with Hoechst 33342 (1:5000; Thermo #H3570), washed in PBST, and mounted in Fluoromount-G® (Southern Biotech #0100–01) with coverslips (Corning #2980-245).

### Cuprizone model

Male C57Bl/6J mice (aged 8 weeks) were fed 0.2% cuprizone-laced diet (Envigo Diet No. 140800) for 3 weeks to induce demyelination[34], followed by 2 weeks of normal rodent diet. Mice were killed at 5-week post-cuprizone during maximal demyelination phase for subsequent CLARITY tissue processing.

### CLARITY tissue processing and immunostaining

Mice were euthanized at day 8 p.i., brains were harvested, bisected, and post-fixed in PBS containing 4% PFA (w/v) overnight at 4 °C. Halved brains were then incubated in PBS overnight at 4 °C. Next, 1-mm sagittal sections were generated using a sagittal mouse brain matrix (Kent Scientific). Each section was then submerged in hydrogel solution (modified from Epp et al. 2015) [35] containing final concentrations of 3% acrylamide (Bio-Rad #161-0140), 3% formaldehyde (Electron Microscopy Sciences #19200), and 0.25% VA-044 thermal initiator (m/v; Wako Chemicals #NC0632395) for 24–48 h at 4 °C. Incubated tissues were polymerized in hydrogel solution at -90 kPa for 3 h at 37 °C, washed three times with PBS, then actively cleared by electrophoresis using X-CLARITY™ Tissue Clearing System (Logos Biosystems). After washing overnight in PBS to remove any residual SDS, the tissues were incubated in PBS containing anti-proteolipid protein (PLP) (clone AA3; hybridoma solution diluted 1/20), 0.1% Triton-X 100 (v/v; Sigma Aldrich #T8787; PBST), 2% goat serum (Abcam #ab7481), and 0.01% sodium azide (Sigma Aldrich #S2002) for 3 days at 50 rpm and 37 °C on an orbital shaker (Thermo Scientific Max Q 4000). After washing for 24 h in PBST, tissues were stained with goat anti-rat IgG (H + L) cross-adsorbed Alexa Fluor 594 secondary antibody (diluted 1:100 in PBST, Thermo Scientific #A-11007) under the same conditions as the primary step and subsequently washed for 24 h in PBST. Tissues were then incubated in X-CLARITY mounting solution (RI = 1.46, Logos Biosystems #C13101) for 24–48 h before mounting between coverslips (Corning #2980-245) using iSpacers® (SunJin Lab).

### Imaris 3D modeling and analysis

Sets of 100 serial images at 1 µm steps in the Z direction, 0.48 µm/pixel in the X, Y-plane, and 0.95 µsec pixel dwell time were acquired with the Zeiss LSM 710 Confocal Microscope and 10 × objective, resulting in whole datasets of 850 × 850 µm in the X, Y-plane and 100 µm in the Z direction. Three-dimensional (3D) images were rendered with Imaris 9.3 software (Bitplane, Oxford Instruments) and analyzed with the software’s automated Filament Tracer module. All images were analyzed in the same fashion by a rater blinded to condition. Creation parameters were as follows:
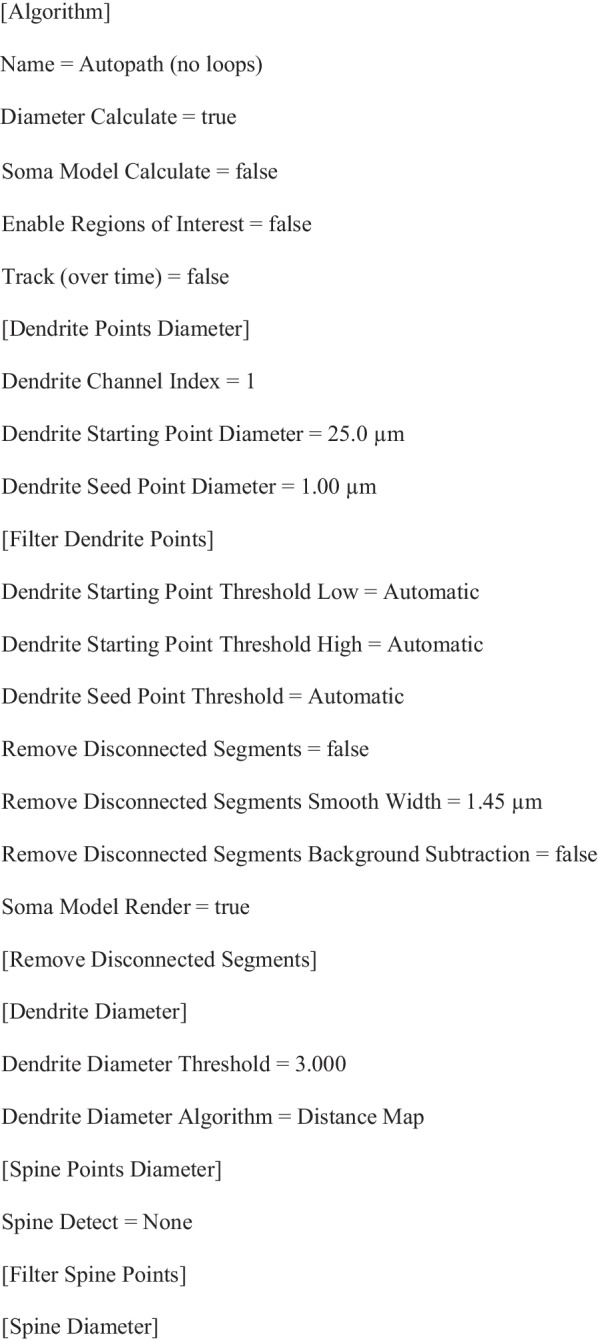


### Myelin extraction

Animals were euthanized via CO_2_ asphyxiation and perfused intracardially with sterile PBS. Whole brains were harvested and immediately flash frozen under liquid nitrogen. Myelin was extracted from brains under sucrose density centrifugation and osmotic shock as previously described [36]. All solvents were prepared fresh at 4 °C, and extractions conducted under ice. Additionally, all extractions were conducted using the same solvents on the same day. Briefly, brains were homogenized with a dounce homogenizer in 0.3 M sucrose containing 0.7 M Tris–HCl (pH 7.4), 10 μg/ml leupeptin (Sigma Aldrich, No. 103476–89-7), 10 μg/ml antipain (Sigma Aldrich, No. 37691-11-5), and 100 μM phenylmethylsulfonyl fluoride (GoldBio, No. P-470). Homogenized brain samples were layered on 0.83 M sucrose containing Tris–HCl, leupeptin, antipain and phenylmethylsulfonyl fluoride (PMSF). The dounce homogenizer was washed twice with 0.3 M sucrose and additionally layered. Samples were centrifuged for 30 min at 75,000 × *g* at 4 °C, and without brakes to prevent disruption of density gradient layers. Following centrifugation, the crude myelin interface between 0.83 M sucrose and 0.3 M sucrose was collected and transferred to a new centrifuge tube containing MQ H_2_O for centrifugation at 75,000 × *g* at 4 °C for 30 m. Osmotic shocks were then conducted twice by resuspending myelin in 5 ml MQ H_2_O and homogenizing thoroughly. Following a 10 min incubation on ice, samples were centrifuged for 15 min at 12,000 × *g*, 4 °C and without brakes. After two osmotic shocks, pelleted crude myelin was resuspended in 2 ml 0.83 M sucrose and layered on 16 ml 0.83 M sucrose, followed by a 0.3 M sucrose. The samples were centrifuged for 15 min at 75,000 × *g*, 4 °C and without brakes. The interface was then transferred to a new tube and resuspended in MQ H_2_O for final centrifugation at 75,000 × *g*, 4 °C and without brakes. The supernatants were carefully discarded to obtain the purified myelin. The purified myelin was then resuspended in 100 μL 1 M Tris–HCl and stored in -80 °C.

### Lipid extraction, lipidomics and data analysis

Total lipids were extracted using the Bligh and Dyer method [37]. Briefly, lipid samples were added to 750 μL 1:2 CHCl_3_:MeOH and vortexed for 15 min at 25 °C. Following 5 min incubation on ice, 250 μL CHCl_3_ and 250 μL MQ H_2_O were added to the sample. Samples were then vortexed for 5 min at 25 °C, and centrifuged for 5 min at 800 × *g* at 4 °C. The organic layer was collected and dried under steady flow of N_2_ gas and resuspended in 90% ethanol for analysis via LC–MSMS. The samples were spiked with 5 μL of 50 μg/ml internal standard mixture (ceramide 18:1/12:0; phosphatidylcholine 12:0/12:0; phosphatidylethanolamine 14:0/14:0; phosphatidylglycerol 14:0/14:0; phosphatidylserine 14:0/14:0) before instrument injection. The samples were analyzed at the Metabolomics Laboratory of the Roy J. Carver Biotechnology Center of the University of Illinois at Urbana-Champaign. Samples were injected into the Dionex Ultimate 3000 series HPLC system (Thermo Scientific, Germering, Germany) which included an autosampler, degasser, and a binary pump. Liquid chromatography separation was conducted on a Thermo Scientific Accucore C18 column (2.1 × 150 mm, 2.6 μm) with a flow rate of 0.4 ml/min. Mobile phase A (60% acetonitrile: 40% H_2_O containing 10 mM ammonium formate and 0.1% formic acid) and mobile phase B (90% isopropanol: 10% acetonitrile containing 10 mM ammonium formate and 0.1% formic acid) was utilized. The detailed method was similar to a previously published paper from the laboratory [38]. The following linear gradient was used: 0 min, 70% A; 4 min, 55% A; 12 min, 35% A; 18 min: 15% A, 20–25 min: 0% A, 26–33 min: 70% A. The injection volume was 10 μL, autosampler was set to 15 °C, and column was kept at 45 °C. Thermo Scientific Q-Exactive MS system (Bremen, Germany) was used for obtaining tandem mass spectrometry under both positive (sheath gas flow rate, 50; aux gas flow rate, 13; sweep gas flow rate, 3; spray voltage, 3.5 kV; capillary temp, 263 °C; aux gas heater temp, 425 °C) and negative electrospray ionization (sheath gas flow rate, 50; aux gas flow rate, 13; sweep gas flow rate, 3; spray voltage, − 2.5 kV; capillary temp, 263 °C; aux gas heater temp, 425 °C). The full scan mass-spectrum resolution was set to 70,000 resolution at *m/z* 200 with scan range of *m/z* 230–1,600. The automatic gain control target was 1,000,000 using the maximum injection time of 200 ms. For MSMS, the mass spectrum resolution was set to 17,500. The automatic gain control (AGC) target was 50,000 with a maximum injection time of 50 ms. Loop count was 10. Isolation window was *m/z* with normalized collision energy (NCE) of 25 and 30 eV. The analysis of lipids in the mPFC via LC–MSMS had pooled samples of 2–3 animals per sample to generate *n* = 4 for PBS, *n* = 4 for IAV at day 8 p.i., and *n* = 3 for IAV at day 16. The analysis of lipids from the isolated myelin of the whole brain via LC–MSMS were from samples that were not pooled, for *n* = 12 saline and *n* = 11 IAV.

Lipids were identified with Thermo Scientific software LipidSearch (Version 4.1.30) as previously described[38]. The lipid signals were normalized to whole brain mass and the corresponding internal standard signal responses. For lipid classes without a corresponding internal standard, positive lipid ion signals were normalized to the signal of ceramide 18:1/12:0 and negative lipid ion species normalized to the signal of phosphatidylglycerol 14:0/14:0. We only focused on monoisotopic species. LipidSearch software was used to predict the possible fragment ions for lipid species within the precursor ion tolerance using the accurate *m/z* values of the precursor ion. The LipidSearch database is built using the known fragment ions for lipid classes and the intensity pattern based on measured spectra. Identification was done by using the parent ion that was based on the accurate mass of precursor ion with the mass shift tolerance of 5 ppm. Identification of the product ion was based on the accurate mass of precursor ion with mass shift tolerance of 8 ppm and MS spectral pattern.

### Total cholesterol assay

As cholesterol is predominantly stored in the esterified form and LC–MSMS detection of cholesterol can be limited to free cholesterol, total cholesterol was measured. mPFC and purified myelin from the whole brain was obtained as previously described. Lipids were extracted using the Bligh and Dyer method, and cholesterol measured via the Cholesterol Amplex Red Assay (Thermo Scientific, #A12216) according to manufacturer’s instructions. Specifically, cholesterol in the samples were hydrolyzed by cholesterol esterase, then oxidized by cholesterol oxidase to yield corresponding ketone and hydrogen peroxide. Hydrogen peroxide was then detected using Amplex Red reagent and horseradish peroxidase. Fluorescent resorufin was measured with excitation of 540 nm and emission of 590 nm. The analysis of cholesterol in the mPFC via Amplex Red Assay was performed on samples from individual mice (*n* = 8 saline and *n* = 10 IAV). Likewise, analysis of cholesterol in the brain was performed on samples from individual mice (*n* = 8 saline and *n* = 10 IAV).

### GW2580 treatment

C57BL/6J mice were housed in reverse light-cycle conditions and were fed a diet of normal chow and water consumption ad libitum prior the treatment. Mice were treated daily with GW2580 (80 mg/kg/d; LC Labs #G-5903) diluted in 200 µl of 0.1% Tween-80, 0.5% hydroxymethyl propyl-cellulose or vehicle alone by oral gavage using plastic feeding tubes (Instech #FTP-18-30-50). Treatment began 7 days prior to inoculation with PBS or influenza and continued for the duration of the study (to day 8 p.i.). In total mice were treated for 15 days. Animal weights were recorded daily.

### Flow cytometry

Flow cytometry was also used to determine the effects of infection on expression of oligodendrocyte specific proteins. As before, mice were euthanized by CO_2_ asphyxiation and perfused with 30 ml of sterile PBS. Brains were extracted, chopped with a sterile razor blade then digested using Neural Tissue Dissociation Kit (Miltenyi #130-092-628) and following manufacturer’s instructions for manual dissociation. The tissue was then passed through a sterile 70-µm filter and pelleted through sterile PBS containing 30% Percoll by centrifugation at 2000x*g* for 20 min. at 4 °C. The cell pellet was suspended in ice-cold flow cytometry staining buffer at a concentration of 1 × 10^6^ cells per 100 µl and Fc receptors blocked for 10 min by incubating samples with anti-CD16/32 (Clone 93; Thermo #14–0161-82). After blocking, cells were stained with fixable viability dye (eFluor 780; Thermo #65-0865-14) and antibodies to the surface marker CD45 (PE; clone 30-F11; Thermo #A16325) for 20 min on ice. Cells were fixed and permeabilized overnight at 4 °C with Foxp3 Transcription Factor Fix/Perm Buffer (Invitrogen #00-5523) and then stained with Alexa Flour 488 conjugated mouse anti-MAG (Clone 513; Sigma #MAB1567A4) as well as unconjugated rabbit anti-Sox10 (Clone SP267; Abcam #ab227680) and rat anti-PLP (Clone AA3; Sigma #MABN2620) for 1 h on ice. After washing, cells were stained with Alexa Fluor 405 conjugated anti-rabbit (Thermo #A-31556) and Alexa Fluor 594 conjugated anti-rat (Thermo # A-11007) antibodies for 1 h on ice. After washing, cells were resuspended in staining buffer and data were acquired on an Attune cytometer (Thermo). Gates were determined using unstained cells, cells incubated with secondary antibodies alone and fluorescence minus one. Results were analyzed using FlowJo software (ver. 10.6.2).

### Primary glial cultures and conditioned medium experiments

Primary microglia and oligodendrocyte cultures were isolated from mixed glial cultures obtained from the forebrains of Sprague Dawley rats previously described[39, 40]. After mixed glial cultures reached confluence, microglia were isolated by shaking the culture in 75 cm^2^ culture flasks for 1 h at 170 rpm and 37ºC. OLs were isolated from the astrocyte layer by shaking overnight at 200 rpm and 37ºC, and incubating in 100 cm^2^ petri dishes at 37ºC for 2 rounds of 1 h each to remove residual contaminating microglia. Primary OL cultures were generated by initially seeding cells into poly-DL-ornithine-coated 24-well plates at a density of 5 × 10^4^ cells per well and grown to confluence in growth medium. OL growth medium consisted of equal parts Dulbecco’s modified eagle medium with 4.5 g/L glucose, L-glutamine, and sodium pyruvate (DMEM; Corning #10–013-CV) and Neurobasal medium (Gibco #21103049) supplemented with penicillin (50 U/ml) and streptomycin (50 µg/ml; Gibco #15140-122), B-27 supplement (2%; Thermo #17504044), N-2 supplement (1%; Thermo #17502048), N-acetyl cysteine (5 µg/ml; Sigma # A8199), D-biotin (10 nM; Sigma #B4639), as well as 10 ng/ml of FGF-basic (Peprotech #100-18B) and PDGF-AA (Peprotech #100-13A). Upon reaching confluence, growth medium was removed and cells were stimulated with microglia-conditioned medium. For conditioned medium, microglia were seeded into 24-well plates at a density of 3 × 10^5^ cells per well in DMEM supplemented with 10% FBS (R&D Systems #S11150H), penicillin (100 U/ml) and streptomycin (100 µg/ml; Gibco #15140-122) and allowed to adhere for 1–2 h prior to stimulation with plain media or media containing LPS (100 ng/ml; # Sigma, St. Louis, MO, USA). To control for potential effects of residual LPS on OL gene expression, control media or media containing LPS was cultured in wells devoid of microglia. After 24 h of stimulation at 37ºC and 5% CO_2_, medium was removed from microglia cultures and clarified by centrifugation at 1000 × *g* for 5 min at 4 ºC. For the 5z condition, OL monocultures were treated with the TAK1-specific inhibitor (5Z)-7-oxozeaenol (Calbiochem #499610) at a concentration of 500 nM for 30 min prior to stimulation with microglia-conditioned medium, to which 5z was added for a final concentration of 500 nM immediately prior to applying to OL monocultures. In all cases, microglia-conditioned medium was used immediately to stimulate primary OL monocultures for 8 h at 37 ºC and 5% CO_2_, then media were collected to measure TNF levels by ELISA according to manufacturer’s instructions (Thermo # KRC3011). RNA was isolated from cells and converted into cDNA as described above in RT-qPCR methods, and expression of *Ugt8* determined as described above using *Actb* as a housekeeping gene. Primers and probes sequences for *Ugt8* and *Actb* are provided (Table 1). Cytotoxicity was assessed by measuring lactate dehydrogenase activity by colorimetric assay (Roche #11644793001) in microglia-conditioned medium as well as medium collected from OL monocultures after stimulation.

### Statistical testing

For lipidomics, differential expression analysis between PBS and IAV was carried out using the two-sided non-parametric Wilcoxon rank sum test. Python SciPy package was used for calculating the rank sum statistic for each lipid species. Expression fold-change of IAV versus PBS was computed as a second measure for evaluating differential expression. We required a *P*-value < 0.05 jointly with fold change > 2 or fold change < 0.5 for significant differential expression. Since only a small number of species were characterized as differentially expressed lipids, the Type 1 error is small and therefore we did not use any *P*-value correction method and worked with *P*-values directly obtained from statistical testing.

Imaris data were analyzed by Kruskal–Wallis one-way ANOVA with Dunn’s multiple comparison test. Gene expression data from the GW2580 experiment were analyzed by two-way ANOVA with Bonferroni multiple comparison test. For all other analyses, two-tailed Student’s t-test was performed between PBS and IAV groups. A *P*-value < 0.05 was considered statistically significant.

## Results

### Transcripts associated with myelination were temporally suppressed by influenza infection.

**Fig. 1:**
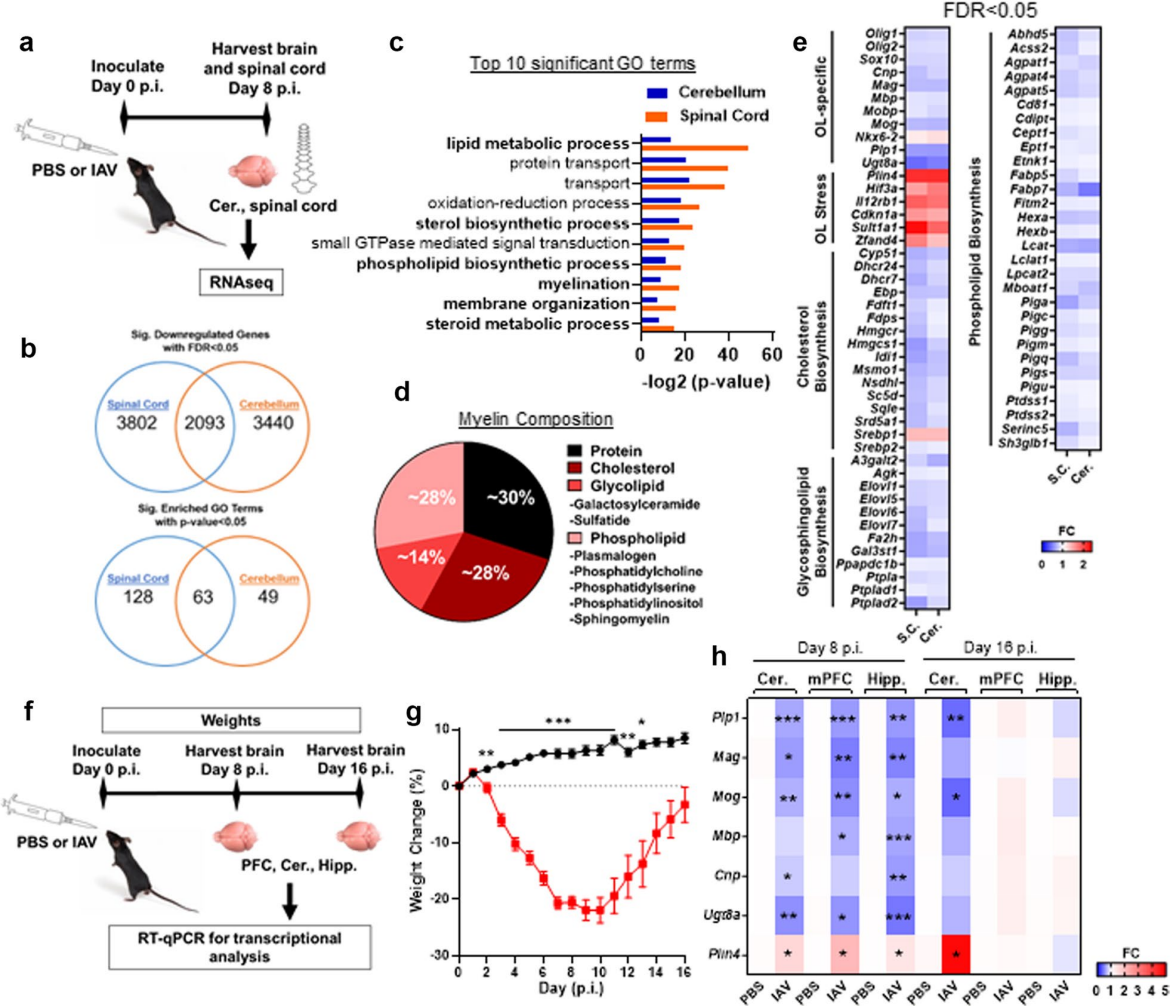
HYPERLINK "sps:id::fig1||locator::gr1||MediaObject::0" Influenza infection temporally downregulates transcripts involved in lipid synthesis and myelination in multiple regions of the CNS. **a** Schematic for bulk RNA-seq experiment. **b** Number of downregulated genes and significantly enriched Gene Ontology (GO) terms in the spinal cord and cerebellum of IAV-inoculated mice compared to PBS-inoculated mice at day 8 p.i. (*n* = 7–8, FDR < 0.05). **c** Fold enrichment of the 25 most highly enriched GO pathways associated with downregulated genes that were shared between the spinal cord and cerebellum at day 8 p.i. **d** Percentage breakdown of major myelin constituents of mouse CNS[41–43]. **e** Expression of genes encoding myelin proteins and enzymes involved in cholesterol biosynthesis, glycosphingolipid biosynthesis, and phospholipid biosynthesis in the spinal cord and cerebellum of infected mice compared to PBS-inoculated controls. Shown in fold change (FC) over control (*n* = 7–8, FDR < 0.05). RNA-seq data are from Blackmore et al. [33]. **f** Schematic for RT-qPCR experiments. **g** Percent daily weight change post-infection (*n* = 18–22 animals per group, pooled from six individual experiments). **h**, **i** RT-qPCR analysis of myelination genes in the cerebellum (Cer.), medial prefrontal cortex (mPFC), and hippocampus (Hipp.) of mice at days 8 and 16 p.i. (*n* = 3–10, Student’s *t* test). Data are presented as mean ± SEM. *P*-value * < 0.05, ** < 0.01, *** < 0.001

We previously demonstrated that influenza A virus (IAV) infection, while confined to the lung, altered the cerebellum and spinal cord transcriptome (Fig. 1a). Our initial analysis of this dataset was focused primarily on genes that were upregulated by infection. Here, using a threshold FDR < 0.05, we identified 2093 downregulated genes in both cerebellum and spinal cord tissues of IAV-infected mice compared to PBS-inoculated controls at day 8 post-infection (p.i.) (Fig. 1b). Gene ontology (GO) enrichment analysis revealed these downregulated genes were associated with 63 GO terms shared between both tissues (Fig. 1b). The most highly enriched terms included *myelination*, *cholesterol biosynthetic process*, *phospholipid biosynthetic process*, and *sphingolipid biosynthetic process*, indicating these lipid biosynthesis pathways might be impaired as a result of IAV infection (Fig. 1c). The downregulation of these pathways is of great interest given that the major lipid classes of myelin in the mouse CNS [41–43] are (1) cholesterol, (2) phospholipid, and (3) glycolipid (Fig. 1d). Indeed, expression of multiple enzymes that are required for cholesterol biosynthesis as well as of several families of lipids involved in phospholipid and glycosphingolipid biosynthesis was decreased in spinal cord and cerebellar tissues of infected mice compared to PBS controls (Fig. 1e). In addition to these lipid biosynthetic enzymes, genes encoding myelin proteins such as *Mag*, *Mog*, and *Plp1* were downregulated in both CNS tissues (Fig. 1e). Moreover, *Plin4*, *Hif3a*, *Il12rb1, Cdkn1a, Sult1a1,* and *Zfand4*, which have recently been identified as markers of disease-specific OLs in an inflammatory, demyelinating mouse model[44] were upregulated in our dataset (Fig. 1e). The effect of infection on the expression of these genes was not affected by mouse sex (Additional file 2: Table S1).

To validate these results and characterize any regional differences in response to infection, we repeated the experiment and measured expression of select genes in the medial prefrontal cortex (mPFC), cerebellum, and hippocampus by RT-qPCR (Fig. 1f). As in previous experiments, infected mice exhibited maximal weight loss by day 8 p.i. (Fig. 1g). At this time point, genes required for cholesterol biosynthesis including *Cyp51*, *Dhcr7*, and *Idi1* were decreased, predominately in the mPFC (Additional file 1: Fig. S1). The OL-specific genes *Plp1*, *Mag*, *Mog*, and *Ugt8a* were also decreased in cerebellum, mPFC, and hippocampus (Fig. 1h). In addition, *Mbp* was decreased in all but cerebellum, and *Cnp* in cerebellum and hippocampus (Fig. 1h). These data validate our previous findings and indicate that IAV infection decreased transcription of OL-specific genes in multiple regions of the brain. Expression of the gene perilipin 4 (*Plin4*), considered here as a proxy for OL stress due to its high expression in disease-state OLs and near absence in healthy conditions[44], was upregulated in all regions (Fig. 1h). By day 16 p.i., body weights returned to baseline, indicating that the mice were recovered from infection (Fig. 1g)[45]. Likewise, OL-specific genes also returned to baseline levels of control mice in the mPFC and hippocampus (Fig. 1i). Interestingly, *Plp1* and *Mog* remained downregulated in the cerebellum while *Plin4* remained upregulated in this region (Fig. 1i). Taken together, these results indicate that IAV infection temporally suppresses the transcription of genes involved in lipid biosynthesis and myelination while upregulating genes previously associated with OL stress.

### Influenza infection altered cellular protein expression without affecting viability

**Fig. 2:**
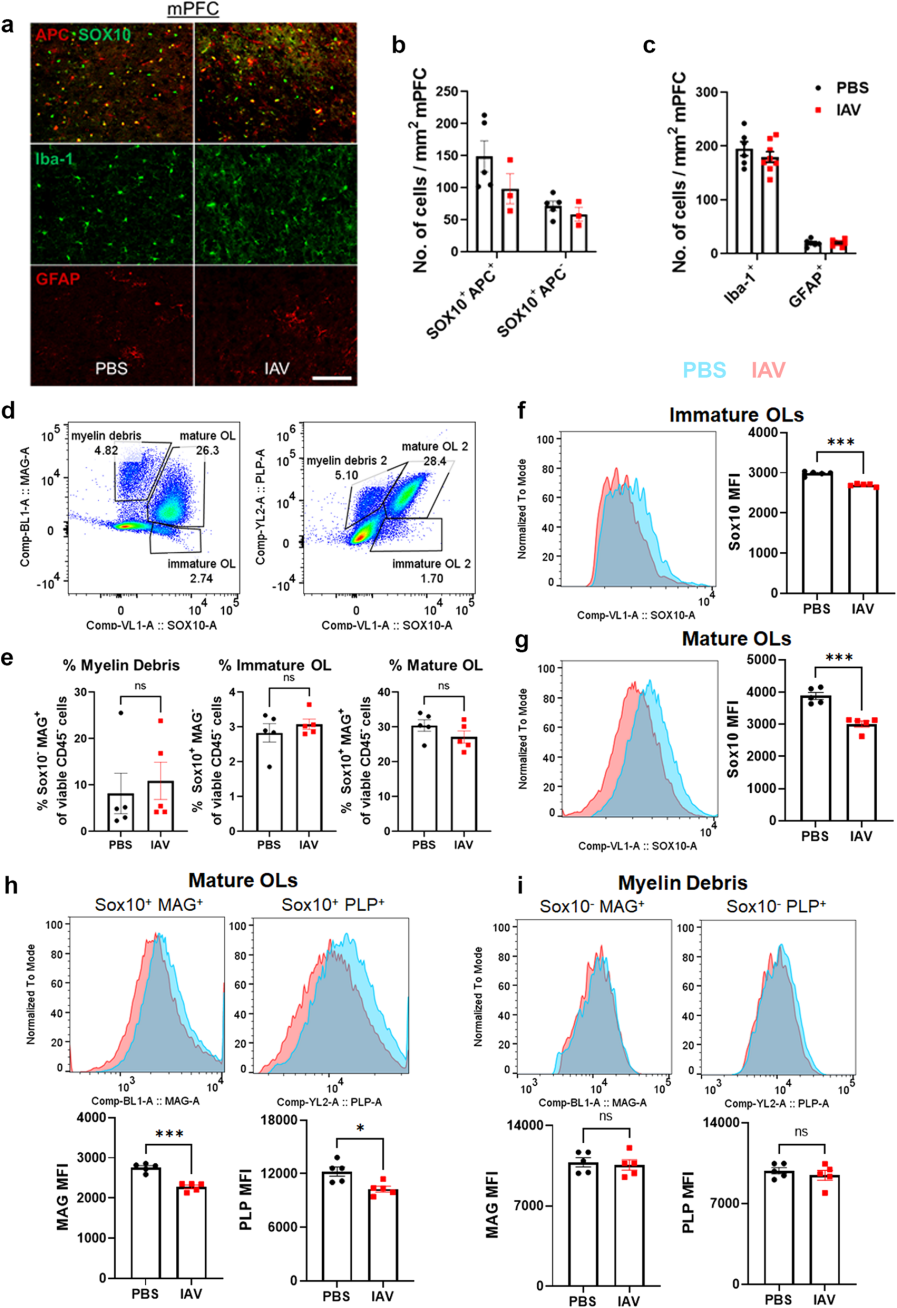
Influenza infection does not affect OL survival but increases marker of OL stress in the mPFC. **a** Representative immunohistochemical staining of mPFC tissue of PBS- and IAV-inoculated mice with anti-APC (CC1 clone), SOX10, Iba-1, and GFAP at day 8 p.i. **b**, **c** Number of SOX10^+^APC^+^ and SOX10^+^APC^−^ cells per mm^2^ mPFC (*n* = 3–5), and number of Iba-1^+^ and GFAP^+^ cells per mm^2^ mPFC (*n* = 6–8) of PBS- and IAV-inoculated mice at day 8 p.i. Flow cytometry was performed on brains of PBS- and IAV-inoculated mice (*n* = 5) at day 8 p.i. to determine percentage of myelin debris (SOX10^−^MAG^+^), immature OLs (SOX10^+^MAG^−^), and mature OLs (SOX10^+^MAG^+^) (**d**, **e**), as well as OL protein expression of immature OLs, mature OLs, and myelin debris (**f**–**i**). Mean fluorescent intensity (MFI) of SOX10 protein expression of immature OLs (**f**) and mature OLs (**g**), MFI of MAG and PLP protein expression of mature OLs (**h**) and myelin debris (**i**). Data analyzed by Student’s *t*-test and presented as mean ± SEM. P-value * < 0.05, *** < 0.001

To assess whether infection had an effect on OL viability and glial activation, we performed immunostaining on brain slices from mice at day 8 p.i. using antibodies specific for OLs (SOX10^+^), mature OLs (SOX10^+^APC^+^), microglia (Iba1^+^) and astrocytes (GFAP^+^). Since changes to myelin genes have been previously reported to occur following chronic stress or social isolation within the mPFC[46, 47], we first focused our analysis on this region. Neither the number of mature (SOX10^+^APC^+^) nor immature (SOX10^+^APC^−^) OLs in the mPFC were affected by infection (Fig. 2a, b). Likewise, the numbers Iba-1^+^ microglia of GFAP^+^ astrocytes within the mPFC were unaffected by infection (Fig. 2c). Although microglia morphology appeared to be altered (Fig. 2a), as has been reported to occur in the hippocampus during infection with the same IAV strain (A/PR8) [31], we observed neither gliosis nor the presence of inflammatory foci, consistent with the non-neurotropic nature of this IAV strain [33, 48, 49]. To confirm that infection did not alter the number of mature or immature OLs and to determine whether OL from infected mice exhibited changes to the expression of myelin proteins, flow cytometry was performed on events isolated from entire brains of PBS- and IAV-inoculated mice at day 8 p.i. The cell isolation procedure generated three distinct populations of events: myelin debris (defined as SOX10^−^PLP^+^ or SOX10^−^MAG^+^ events), immature OLs (defined as SOX10^+^PLP^−^ or SOX10^+^MAG^−^) and mature OLs (defined as SOX10^+^PLP^+^ or SOX10^+^MAG^+^). The percentage of immature and mature OLs did not differ between conditions (Fig. 2d, e). However, mean fluorescence intensity analysis performed on viable immature (SOX10^+^MAG^−^, SOX10^+^PLP^−^), and mature (SOX10^+^MAG^+^, SOX10^+^PLP^+^) OL populations showed that SOX10 expression in OLs from IAV-inoculated mice was slightly reduced in both cell types. Furthermore, infection modestly reduced MAG and PLP levels by 17.4%, and 16.0%, respectively, compared to those from PBS-inoculated controls (Fig. 2f–h), corroborating the mRNA expression analysis (Fig. 1). Myelin proteins are known to have relatively long half-lives [50–53]. As such, small changes in protein expression within the cell may not translate into global changes in protein levels within the myelin sheath. Therefore, we measured mean fluorescent intensity levels of PLP and MAG within the myelin debris fraction. This analysis revealed no differences between PBS- and IAV-inoculated mice (Fig. 2i). Likewise, immunoblots performed on blocks of tissue isolated from mPFC, cerebellum, and hippocampus tissue of PBS- and IAV-inoculated mice indicated that infection did not alter abundance of MAG, MOG, or SOX10 (Additional file 1: Fig. S2a, b). In contrast, PLIN4 expression was increased in all three regions (Additional file 1: Fig. S2c). Furthermore, immunostaining for PLIN4 revealed it was, albeit not exclusively, co-localized to APC^+^ mature OLs and that the number of PLIN4-expressing mature OLs in the mPFC was increased by infection (Additional file 1: Fig. S2d). These data indicate that IAV infection perturbs OL homeostasis, but does so without affecting OL survival.

### Influenza infection did not cause demyelination in the mPFC

To determine if infection caused demyelination we used CLARITY tissue-clearing and PLP staining to generate myelinated fiber scaffolds of the mPFC (Additional file 1: Fig. S3a). To ensure changes to myelin levels could be detected by this method as well as to identify which output measures from Imaris™ were indicative of demyelination, we analyzed mPFC tissue of mice at 5 weeks post-cuprizone intoxication, which is known to cause demyelination in this region[54]. The resulting 3D models of the three treatment groups (Additional file 1: Fig. S3b) produced quantifiable attributes related to myelinated fibers such as filament diameter and volume (Additional file 1: Fig. S3c; Additional file 3: Tables S2, Additional file 4: Table S3). Compared to PBS-inoculated mice, cuprizone-fed mice had smaller values for mean filament diameter, a reflection of myelin thickness, as well as decreased total filament volume, which is indicative of total myelin (Additional file 1: Fig. S3c). However, we found no differences between the PBS- and IAV-inoculated mice at day 8 p.i. (Additional file 1: Fig. S3c, Additional file 4: Table S3), indicating that infection did not cause demyelination of this region.

### Influenza infection altered the myelin lipidome

**Fig. 3:**
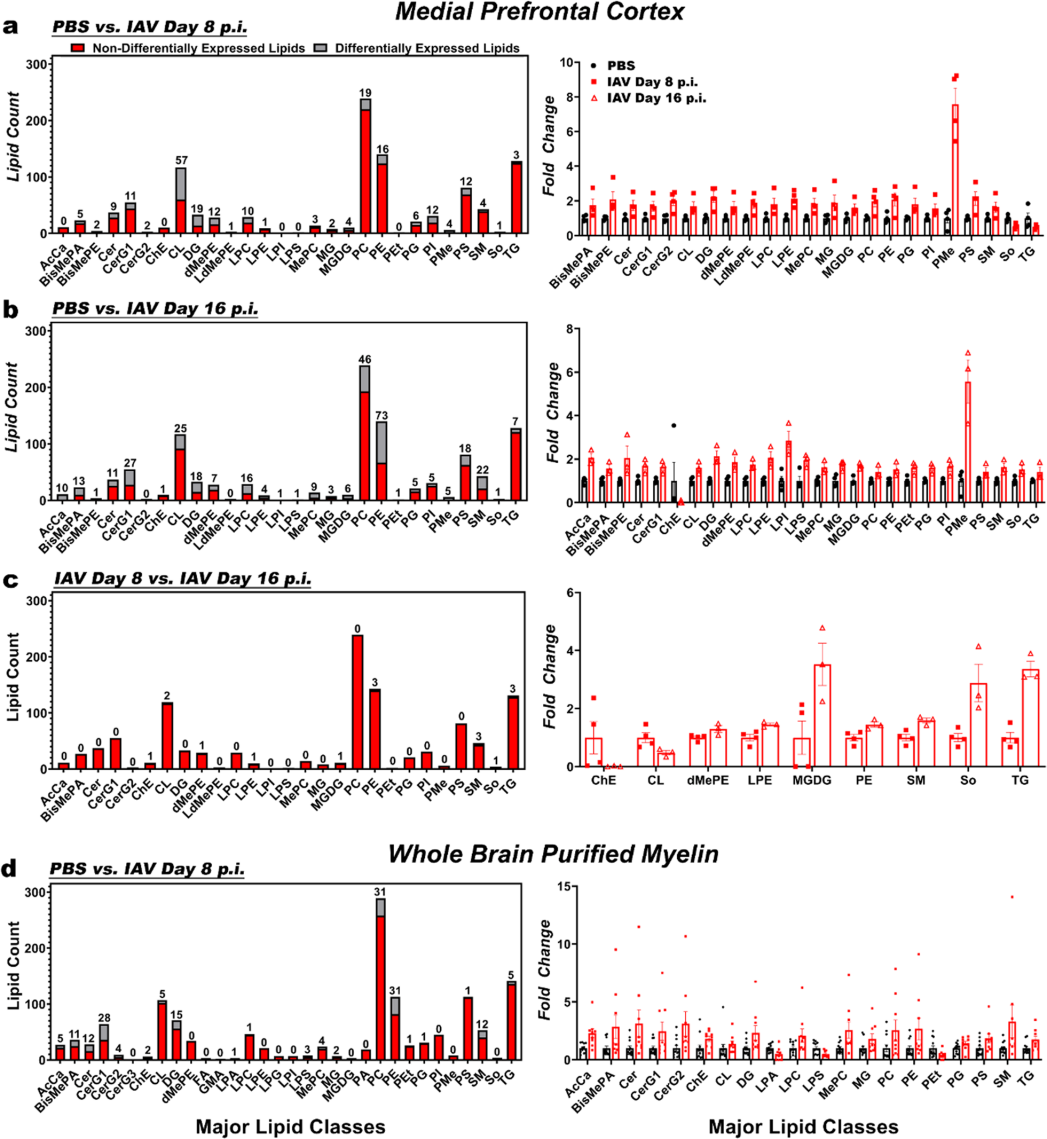
Lipidomics analyses reveal lipid changes in purified myelin. **a**–**c** Left: differential expression of lipid species between PBS-inoculated mice (**a**, 2–3 animals pooled, *n* = 4) and IAV-inoculated mice at day 8 p.i. (**a**, 2–3 animals pooled, *n* = 4 per group), and at day 16 p.i. (**b**, 2–3 animals pooled, *n* = 3 per group) in the mPFC. For analysis of lipids in the mPFC, 2–3 animals were pooled for each representative sample. Values above histogram represent number of individual lipid species unique to its respective major lipid class. Right: differentially expressed lipids (*p* < 0.05) determined by Wilcoxon rank sum test of overall major lipid classes plotted as fold change over PBS-controls (in **a**, **b**) or fold change over IAV-inoculated mice at day 8 p.i. (in **c**). **d** Left: differential expression of lipid species between PBS-inoculated mice (d, unpooled animals, *n* = 12), and IAV-inoculated mice at day 8 p.i. (d, unpooled animals, *n* = 11) in purified myelin isolated from whole brain. Right; Differentially expressed lipids (*p* < 0.05) determined by Wilcoxon rank sum test of overall major lipid classes plotted as fold change over PBS controls

Transcriptomic analyses indicated that infection altered lipid metabolism of OLs (Fig. 1). Therefore, we sought to determine if IAV infection caused changes to the lipidome within the mPFC. We isolated the mPFC from PBS- or IAV-inoculated mice at day 8 and day 16 p.i. In total, 1091 lipid species belonging to 28 major lipid classes were identified in the mPFC. Three pair-wise comparisons were performed: PBS vs. IAV at day 8 p.i. (Fig. 3a), PBS vs. IAV at day 16 p.i. (Fig. 3b), and IAV at day 8 p.i. vs IAV at day 16 p.i. (Fig. 3c). Fold change of differentially expressed lipid species within each class is provided in Additional file 5: Tables S4, Additional file 7: Table S6. In the mPFC at day 8 p.i., there were a total of 215 lipid species that were differentially expressed in IAV-inoculated mice compared to PBS-inoculated controls (Fig. 3a; Additional file 5: Table S4). While a majority of the major lipid classes increased as a result of IAV infection at day 8 p.i., three lipid species belonging to So and TG classes were decreased (Fig. 3a). At day 16 p.i., 338 lipid species belonging to 25 major lipid classes were differentially expressed because of infection (Fig. 3b; Additional file 6: Table S5). Interestingly, the only major lipid class that was not increased by IAV inoculation at day 16 p.i. in the mPFC was cholesterol. Notably, there were minimal differences observed when comparing changes to lipid species between IAV-inoculated mice at day 8 p.i. and day 16 p.i. (Fig. 3c; Additional file 6: Table S6). Specifically, only 16 lipid species were differentially expressed within the mPFC of infected mice between these time points. These data indicate that modulation of lipids by infection does not recover to baseline levels by day 16 p.i., despite that mice had recovered from infection and OL-specific genes were found to have returned to normal expression levels by this time point (Fig. 1).

To further assess the effects of IAV infection on myelin, we repeated the experiment in order to perform lipidomic analysis on myelin purified from the whole brain by density gradient fractionation and osmotic shock. Myelin enrichment was verified by SDS-PAGE and western blot, which confirmed abundant PLP and negligible levels of the astrocyte protein GFAP in extracted myelin fractions (Additional file 1: Fig. S5). A total of 176 lipid species were differentially expressed out of 1356 total lipid species identified (Fig. 3d; Additional file 8: Table S7). With the exception of LPA, LPS, and PEt, which decreased by nearly twofold, most of the affected lipid classes were increased in the myelin of IAV-inoculated mice compared to PBS controls at day 8 p.i. (Fig. 3d). These data corroborate the lipidomic findings from the mPFC, where many lipid species were increased as a result of infection.

**Fig. 4:**
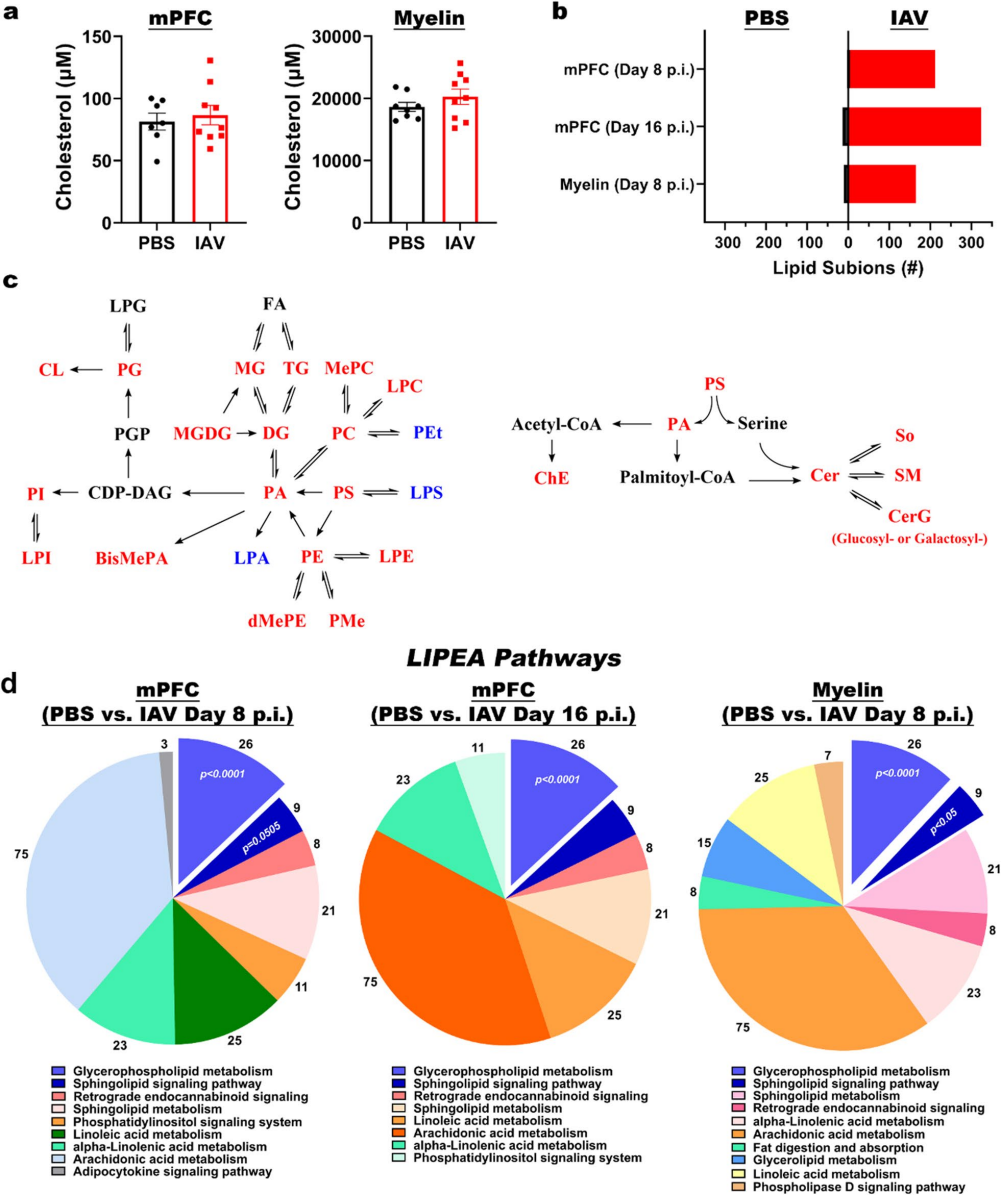
LIPEA bioinformatics reveals changes in glycerophospholipid metabolism. **a** Cholesterol levels in mPFC (unpooled animals, *n* = 8 PBS and *n* = 10 IAV D8) and isolated myelin from whole brain (unpooled animals, *n* = 8 PBS and *n* = 10 IAV D8) in PBS- or IAV-inoculated mice at day 8 p.i. Data are presented as mean ± SEM. **b** Number of differentially expressed lipid sub-ions of PBS- or IAV-inoculated mice in the mPFC at days 8 and 16 p.i., or in purified myelin at day 8 p.i. **c** Schematic summarizing differentially expressed lipid within each class as upregulated (red) or downregulated (blue). **d** Differentially expressed lipids with biological pathways associated with lipid synthesis and degradation analyzed by LIPEA with Bonferroni correction. Values represent number of lipid species involved in each pathway

Both transcriptomic and targeted analyses of genes encoding enzymes involved in cholesterol biosynthesis showed that their expression was decreased by infection (Fig. 1e; Additional file 1: Fig. S1). Cholesterol was not detectible in the mPFC at day 8 p.i. and 1 out of 11 (9.1%) detectible cholesterol sub-ion species was decreased at day 16 p.i. (Fig. 3a–c). In contrast, analysis performed on the myelin fraction demonstrated that 2 of the 6 sub-ion species (33%) within the cholesterol lipid category were increased by infection (Fig. 3d). However, cholesterol detection by LC–MSMS requires de-esterification which can influence the readout of other major lipid classes. Therefore, we replicated the experiment and measured total cholesterol by colorimetric assay. Analysis performed on both the mPFC and purified myelin from brains of PBS and IAV-inoculated mice isolated at day 8 p.i. indicated cholesterol levels were not different between the groups (Fig. 4a).

Lipids known to be involved in the structural integrity of myelin include Cer, CerG, PE, PC, SM and So. All of these were differentially expressed in both mPFC and myelin fractions isolated from the whole brain, with most sub-ions being upregulated (Fig. 4b, c). Therefore, we conducted bioinformatic analysis using LIPEA to determine which lipid pathways were most altered by infection. Following Bonferroni correction, we discovered that glycerophospholipid metabolism and sphingolipid signaling pathways were the most altered by infection (Fig. 4d). Collectively, these data demonstrate that IAV infection caused alterations to the myelin lipidome which persisted at a time point that corresponds to viral clearance from the lung[45].

### Infection-induced changes to OL-specific transcripts were associated with glial activation and were partially attenuated following systemic treatment with a CSF1R antagonist

**Fig. 5:**
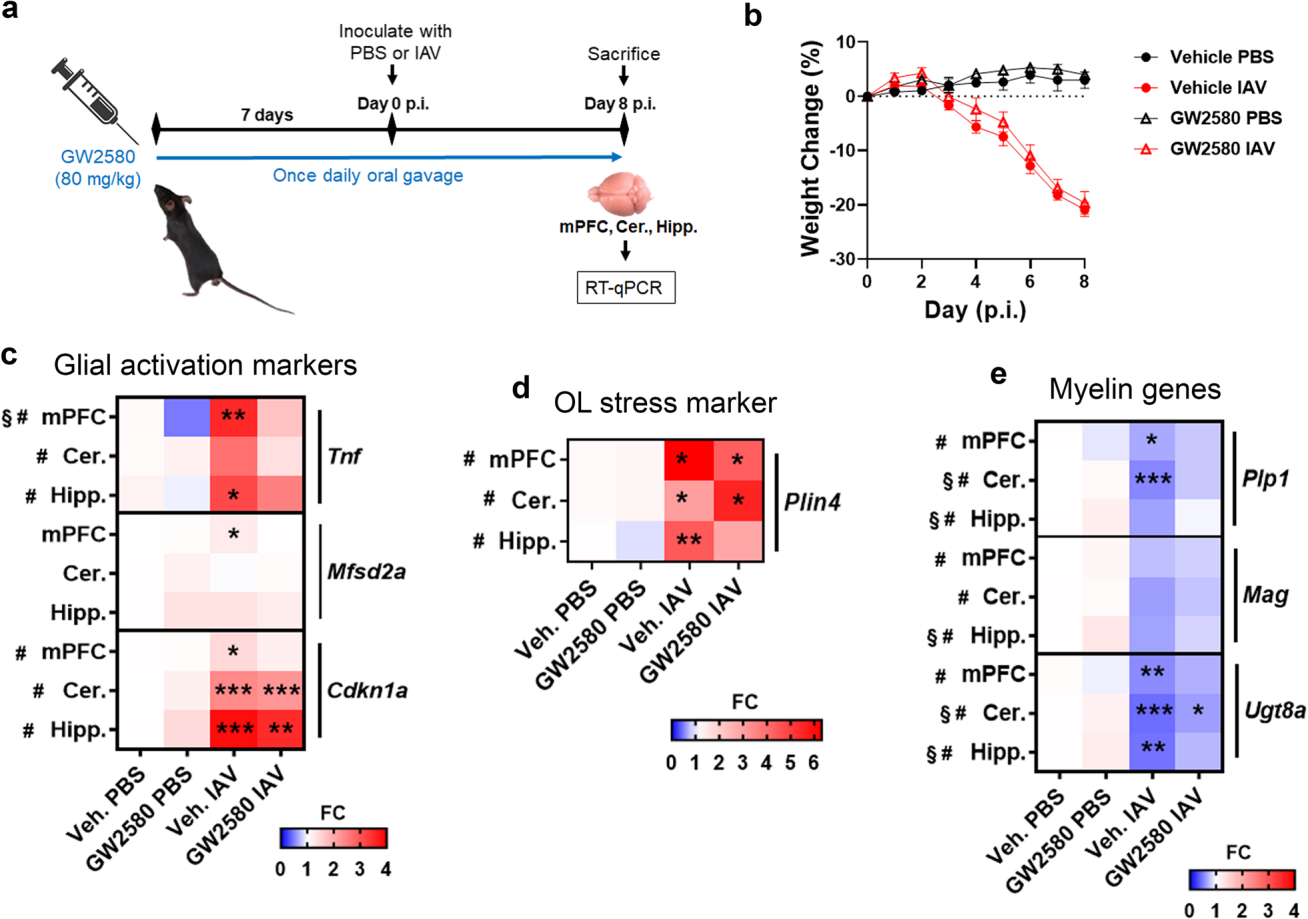
Infection-induced changes to OL-specific transcripts are partially attenuated following treatment with a CSF1R antagonist. **a**–**e** C57BL/6J mice were treated with vehicle or the CSF1R inhibitor GW2580 (80 mg/kg/d) by oral gavage for 7 days then inoculated with PBS or IAV (*n* = 5–6). **b** Percent daily weight change post-infection (*n* = 5–6). Data presented as mean ± SEM. Brain regions were micro-dissected at day 8 p.i. and gene expression analyzed. Effects of infection and treatment on expression of genes associated with glial activation (**c**), OL stress (**d**), and myelination **e** in the medial prefrontal cortex (mPFC), cerebellum (Cer.) and hippocampus (Hipp.) are shown (*n* = 5–6) as fold change (FC) over PBS-control. Significance was determined by two-way ANOVA with Bonferroni correction. Main effect of GW2580 treatment is indicated by the symbol §. Main effect of infection is indicated by the symbol #. *P*-value significance * < 0.05, ** < 0.01, *** < 0.001

Our results indicated that IAV infection altered OL homeostasis. We, and others, have previously shown that peripheral viral infection is associated with glial activation [29, 30, 33, 48], and increased expression of genes such as *Tnf, Il1a* and *C1qa* by microglia in response to peripheral inflammation has been shown to affect other glial subsets [55]. We next questioned whether inhibiting microglial activation might reverse the effect of infection on the transcriptomic changes that occur to OLs. Microglial activation, proliferation and survival are, in part, contingent on constitutive colony stimulating factor receptor (CSF1R) signaling. Indeed, analysis of bulk RNA-seq from cerebellar tissue indicated that *Csf1* was increased at day 8 p.i., whereas expression of *Il34*, was unchanged (Additional file 1: Fig. S5). Therefore, we questioned whether treatment of mice with GW2580, a brain penetrant CSF1R antagonist [58], alleviated changes to OL transcripts resulting from IAV infection (Fig. 5a). Treatment had no effect on weight loss due to infection (Fig. 5b). A pilot study indicated that *Tnf*, *Mfsd2a* and *Cdkn1a* may represent markers of glial activation in response to IAV infection (data not shown). Indeed, infection increased expression of *Cdkn1a* in the mPFC, cerebellum and hippocampus, and of *Tnf* in the mPFC and hippocampus (Fig. 5c). In contrast, *Mfsd2a* expression was only increased in the mPFC (Fig. 5c). Treatment with GW2580 appeared to lower expression of *Tnf* in the mPFC, cerebellum and hippocampus. However, the effect of treatment was only significant for the mPFC (Fig. 5c). As observed in our previous experiments, *Plin4* was upregulated by infection, but its expression was not significantly affected by GW2580 treatment (Fig. 5d). Also as before, the OL-specific genes *Plp1* and *Ugt8a* were downregulated by infection (Fig. 5e). Treatment with GW2580 tended to increase the expression levels of myelin genes, which was revealed by a significant main effect of treatment in both the hippocampus and cerebellum (Fig. 5e). Collectively, these data show IAV infection promotes glial reactivity, which coincides with the suppression of genes that are involved in myelin maintenance. Moreover, antagonism of CSF1R partially inhibited glial reactivity and in part increased expression of OL-specific transcripts.

### Secreted factors from activated microglia suppressed Ugt8 in primary OL cultures

**Fig. 6:**
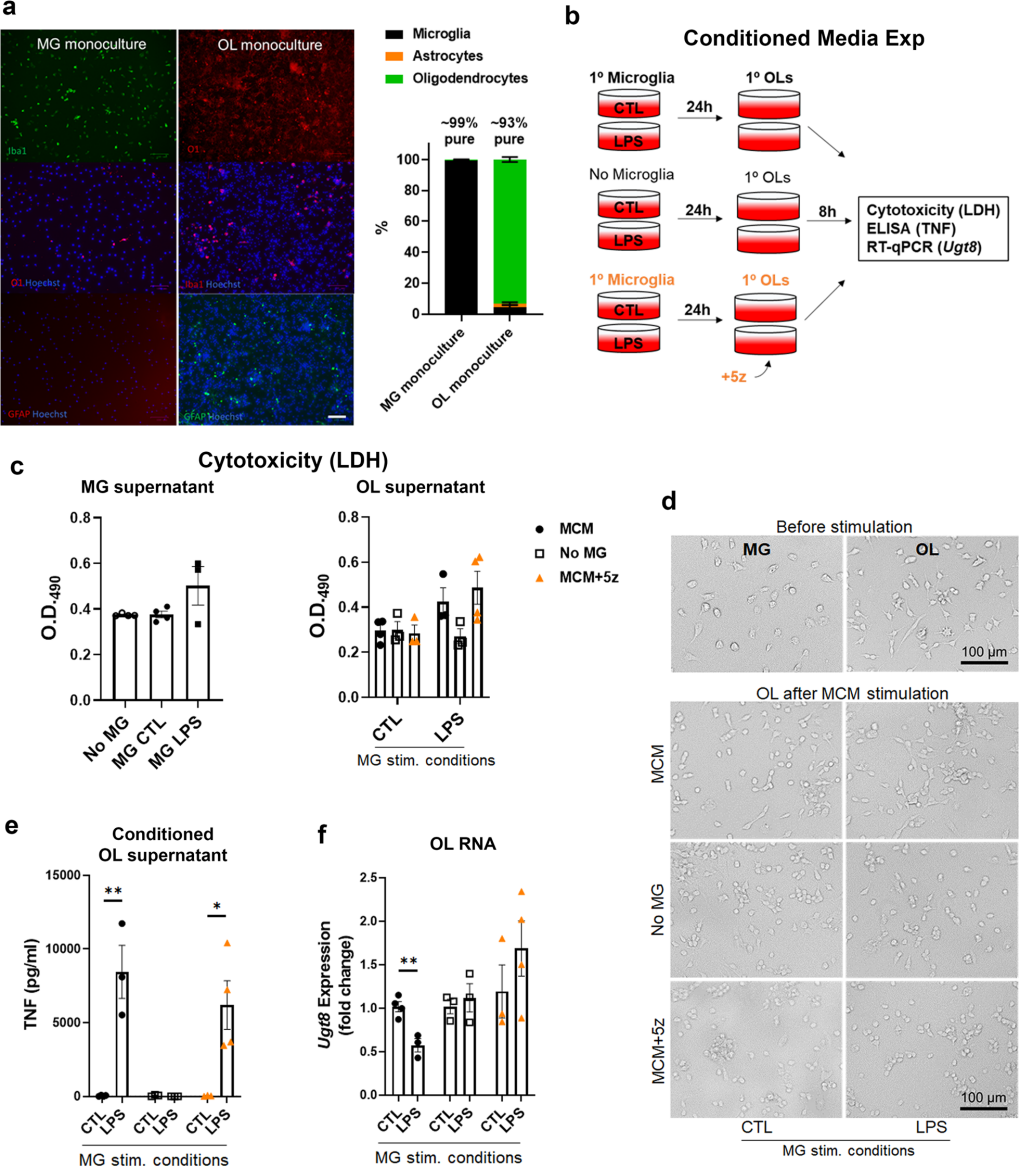
Conditioned media from LPS activated microglia downregulates *Ugt8* expression in OLs cultures. **a** Primary microglia (MG) and oligodendrocytes (OLs) were derived from rat mixed glial cultures and purity of monocultures was determined by staining for markers of microglia (Iba1), oligodendrocytes (O1), astrocytes (GFAP). Hoechst was used to stain nuclei. Data are from the average of five 10 × images per well from three independent cultures (*n* = 3) and are presented as means ± S.E. **b**–**d** Microglia (black circles) or wells devoid of cells (open squares) were cultured with media (CTL) or media containing LPS (100 ng/ml) for 24 h. In some conditions, OL monocultures were pre-treated with the TAK1 inhibitor (5Z)-7-Oxozeaenol (5z; 500 nM) prior to stimulation with microglial-conditioned media containing 5z (500 nM; orange triangles). Primary OL cultures were stimulated with conditioned media for 8 h. **c** Cytotoxicity was assessed by measuring LDH release from microglia-conditioned medium (MG supernatant) as well as medium collected from OL monocultures after stimulation (OL supernatant). **d** Brightfield images of MG and OLs prior to stimulation (top) and OLs after MCM stimulation (bottom). **e** TNF levels of OL supernatant after stimulation as determined by ELISA. **f** OL expression of *Ugt8* as determined by RT-qPCR. Results are combined means ± S.E. from 3–4 independent experiments. Significance was determined by Student’s *t*-test. *P*-value significance * < 0.05, ** < 0.01

The protein UDP glycosyltransferase 8, encoded by the *Ugt8* gene, is almost exclusively expressed in OLs within the brain[56], is responsible for the transfer of galactose to ceramide and is needed for the generation of sulfatide, an important lipid component of myelin. Notably, deletion of *Ugt8a* in mice is characterized by an increase in PE, PS and PC with concurrent lack of sulfatide[57]. We consistently observed suppression of *Ugt8a* (Figs. 1, 5) as well as upregulation of PE, PS and PC lipid species in tissues and myelin from infected mice compared with controls (Fig. 3). Since GW2580 treatment during infection partially reversed the effects of infection on *Ugt8a* expression levels (Fig. 5), we questioned whether secreted factors from activated microglia were capable of directly suppressing *Ugt8* expression in primary OL cultures. The microglia and oligodendrocyte monocultures were found to be approximately 99% and 93% pure, respectively, as determined by immunostaining for lineage-specific markers Iba1 and O1 (Fig. 6a). For these experiments, primary microglia (MG), or wells devoid of microglia (No MG), were incubated with either media (CTL) or media containing the toll-like receptor 4 ligand lipopolysaccharide (LPS) for 24 h (Fig. 6b). Subsequently, conditioned medium from each condition was transferred onto primary OLs. Since transforming growth factor beta activated kinase (TAK)1 signaling facilitates intracellular signaling following receptor ligation of many proinflammatory factors, including TNF and IL-1, we included experiments in which we added the TAK1 inhibitor 5Z-7-oxozeaenol (5z) to microglia-conditioned medium immediately prior to OL challenge. Stimulation of OLs with microglia-conditioned medium did not alter cell viability (Fig. 6c, d), despite that microglia challenged with LPS upregulated TNF compared to vehicle treated controls, confirming their activation status (Fig. 6e). Conditioned medium from LPS treated culture plates containing no microglia did not increase supernatant levels of TNF after it was transferred to OLs. This finding is consistent with a lack of toll-like receptor 4 expression on OLs [56] and indicates that OLs did not secrete TNF in response to residual LPS in conditioned medium (Fig. 6e). Stimulation of OLs with conditioned medium from microglia challenged with LPS suppressed *Ugt8* expression compared to cultures stimulated with conditioned medium from control microglia (Fig. 6f). In contrast, *Ugt8* expression did not differ between OLs treated with control or LPS medium that had been conditioned in the absence of microglia, indicating that reductions in *Ugt8* expression were not attributable to direct effects of LPS on OLs (Fig. 6f). Finally, the addition 5z to microglia-derived LPS conditioned medium before OL challenge abolished the changes in OL *Ugt8* expression that were observed following microglia-derived LPS conditioned medium alone (Fig. 6f). Collectively, these data show that activated microglia produce soluble factors that can act directly on OLs to suppress *Ugt8* expression, and that these factors likely require TAK1 signaling to mediate this effect.

## Discussion

In the current study, we investigated the effect of infection on OL homeostasis. We found infection downregulated many transcripts involved in myelination within the adult mouse CNS. Moreover, infection decreased expression of select proteins at the cellular level and increased expression of PLIN4, a protein previously characterized as a marker of OL stress. Unbiased lipidomic analyses revealed that infection also caused changes to the myelin lipidome and that these changes were predominately characterized by upregulation of lipids, but also entailed downregulation of species belonging to the LPA, LPS, and PEt lipid classes. As reported previously[48] infection increased expression of the proinflammatory cytokine *Tnf*, in a manner indicative of microglia activation and systemic treatment with a CSF1R antagonist partially reversed the effect of infection on OL-specific transcripts. Moreover, we found that conditioned medium from activated microglia was capable of suppressing *Ugt8* in primary OLs and that this effect could be abolished when a TAK1-specific inhibitor was added to the conditioned medium. To our knowledge, this is the first study to demonstrate that a respiratory viral infection is capable of facilitating transcriptomic, proteomic and lipidomic alterations to OLs and myelin.

In recent years, it has become clear that myelin is dynamic and subject to remodeling even in adulthood [12, 58, 59]. Myelin can be remodeled by environmental factors such as learning a new task [13, 14], social isolation [46], and by social defeat stress [60, 61]. Stress is considered a potent risk factor for the onset of depression and mood disorders [62]. In socially isolated or stressed mice, OL and myelin transcripts were decreased in the prefrontal cortex [46, 60, 63], a region that is compromised in depression and other disorders associated with white matter abnormalities. Here, we add to the list of environmental factors that can affect oligodendrocytes by showing that myelin and cholesterol biosynthesis transcripts were decreased in the mPFC, cerebellum, and hippocampus of influenza A virus-infected mice without causing reductions in OL viability. We also show that infection altered the lipidome within the mPFC and of purified brain myelin.

Despite that infected mice exhibited transcriptional changes to myelin genes and lipidomic changes to brain and myelin tissues, immunofluorescence and immunoblotting analysis indicated that these effects occurred in the absence of gross changes to myelin proteins. Given the qualitative nature of the immunoblotting technique, these findings are not surprising, as an appreciable reduction in myelin proteins by this technique would likely be more indicative of demyelination rather than myelin remodeling. In fact, it has been shown previously that the myelin lipidome can be altered without affecting the levels of myelin proteins [19, 57] or myelin ultrastructure [57], which is in line with the data herein. Nevertheless, even in the absence of overt structural changes, alterations to the biochemical composition of myelin can have functional consequences such as decreased conduction velocity [64]. Because myelin proteins are long-lived with half-lives on the order of months [50–53], it is reasonable to conclude that downregulation of genes such as *Plp1, Mag,* and *Mbp* does not immediately translate into significant global changes to myelin proteins in infected mice at day 8 p.i., especially at regions distal to the oligodendroglia soma. Notably, PLP is a major constituent of compact myelin, which turns over at a slower rate than noncompact myelin [65]. Likewise, MAG is transported to periaxonal membranes of myelin internodes where it supports paranodal formation, axon cytoskeletal arrangement, and maintenance of the periaxonal space [66–69]. As such, decreases in mRNA levels may be initially correlated with proteins levels within the soma. Our flow cytometry results lend support for this hypothesis. In contrast, PLIN4, a marker of cellular stress in OLs, was upregulated in IAV-inoculated mice where it was found to co-localize to APC^+^ mature OLs, among others. These data suggest that OL proteins and lipid species that comprise myelin are differentially impacted by environmental factors such as infection and underscore the importance of evaluating changes to myelin proteins within the myelin itself as well as the cell body and in conjunction with lipidomic analyses for a more comprehensive understanding of how these factors might contribute to myelin remodeling.

By lipidomic analysis, we found that approximately 13% of total lipid species identified were affected by IAV infection, the majority of which were upregulated. Although RNA analyses revealed the transcription of genes involved in lipid biosynthetic pathways was downregulated, it is important to recognize that most lipids are often short-lived and precursors are continuously recycled [70, 71]. Thus, downregulation of an enzyme involved in a lipid biosynthetic pathway may cause a bottleneck accumulation of certain lipid substrates. The structural integrity of myelin is dependent on cholesterol, ceramides, glucosyl- or galactosyl-ceramides, and phosphatidylethanolamines. In conjunction with proteins, these lipids participate in formation of compact myelin [72, 73]. It is plausible that the observed increase of lipids with known properties of compaction occurs as a natural means to compensate for changes to OL homeostasis. Regardless, the accumulation of ceramide and sphingomyelin species in conjunction with suppression of *Ugt8a*, is noteworthy since others previously reported that brain ceramide levels were increased in mice that lack *Ugt8a*, while glycolipids (i.e., galactosylceramide) and sulfatide were diminished [57]. In the same study, mass spectroscopy analysis indicated that *Ugt8a* mutant mice have an approximate 20% increase in phosphatidylethanolamine (PE), phosphatidylserine (PS) and phosphatidylcholine (PC) compared to wild-type controls. As such, suppression of *Ugt8a* by infection might underlie our observation that select ceramide and sphingomyelin species were accumulated in myelin. Additionally, infection-induced increase in PLIN4, a protein essential to lipid droplet stabilization [74], may reflect increased cellular accumulation of lipid. The data herein are the first to demonstrate the capacity for IAV infection to alter the myelin lipidome, which persisted even at time point that corresponds to complete clearance of infectious virus from the periphery [45]. How long these changes persist and in which anatomical regions and/or white matter tracks specifically, remain to be determined but may be of relevance when considering the pathogenesis of virus-induced neuropsychiatric conditions, as occurs in some IAV and SARS-CoV-2 patients.

Cholesterol is a critical rate-limiting factor for myelin biosynthesis [75]. While shotgun lipidomics has the advantage of being high throughput, it is difficult to delineate isobars using this technique [76]. Additionally, cholesterol that is often stored in their esterified form in the membranes cannot be reliably detected by LC–MSMS without a de-esterification process that may cause other changes in the lipids extracted from myelin. Our results align with previously published work indicating that myelin extraction from 8- to 10-week-old mice analyzed via LC–MSMS did not yield high levels of detectable cholesterol [77]. Nevertheless, total cholesterol measurements indicated that infection did not affect levels either in the mPFC or within purified brain myelin. As with protein levels, the disconnect between suppression of genes involved in cholesterol production and cholesterol levels within myelin is likely attributable to its high abundance and stability.

There are some caveats to current study. For instance, our initial observations on OL gene changes resulting from infection indicated that there were no sex differences and thus male mice were used for subsequent studies. Whether there are sex differences in gene expression across brain regions or differential changes to the lipidome by infection remain to be determined. Another caveat of the current study is that we did not measure changes to the myelin lipidome across brain regions or in different myelin tracts that result from infection, which may exhibit differences in magnitude and/or temporal expression. Finally, analysis of our bulk-seq dataset indicated that the CSF1-CSF1R axis might represent a potential target for controlling microglia activation in response to systemic inflammation, and thus provided the basis for assessing the effects of a CSF1R antagonist on transcriptomic changes to OLs. That IAV-induced changes to OL-specific transcripts were partially reversed by antagonizing CSF1R may implicate a role for activated microglia in this process. Since microglia were not deleted by treatment, it is possible, that their activation accounts for all changes to OL homeostasis either directly or indirectly through astrocyte activation [55]. Alternatively, GW2580 treatment during infection may have reduced peripheral inflammation and that this immunosuppression affected OLs in either a cell-autonomous or non-cell autonomous fashion [78]. However, the findings that conditioned medium from activated microglia was sufficient to decrease *Ugt8* expression in primary OLs and that this effect was blocked when a TAK1-specific inhibitor was added to OL cultures, lend strong support for a role of microglia-derived factors, including cytokines, such as TNF, in facilitating the observed changes.

Other contributing factors resulting from infection likely influence OL physiology and should not be discounted. For instance, severe respiratory viral infections, as occurs in this model, cause dyspnea and hypoxia. Notably, Verhoeven et al. have shown that SpO_2_ levels can decrease to 80% in mice that received a lethal dose of the same IAV strain that we used in our studies [79]. By comparing our own data to those reported by Verhoeven, we suspect that SpO2 levels in our infected mice are between 85 and 95%. This notion is supported by the observation that IAV-induced upregulation of hypoxia-related genes such as *Hif3a* and *Ddit4* as determined by bulk-seq. That infected mice exhibit sustained hypoxia is of relevance as many studies have shown that hypoxia is determinetal to OLs [80, 81]. Moreover, microglia activation and cellular changes consistent with hypoxia have been used to characterize pattern three type lesions in multiple sclerosis patients [82, 83]. Thus, while we have focused on microglial secreted factors within the conditioned medium experiment it is possible that pneumonia-related hypoxia or “virtual hypoxia” brought on by microglia activation [83] also contribute to altered OL homeostasis. Pertinent to this point, hypoxic conditions are sufficient to activate TAK1 signaling [84]. As such, the contribution of TAK1 activation in mediating disruptions in OL homeostasis as occurs during peripheral viral infection is of interest.

In summary, our results demonstrate that peripheral infection with IAV altered the OL transcriptome and the myelin lipidome in the adult mouse CNS. These effects were partially reversed when IAV-inoculated mice were administered a CSF1R antagonist during the course of infection. Overall, these observations improve our understanding of the capacity of peripheral viral infection to contribute to myelin remodeling. The functional consequence of this phenomenon during health and disease is the subject of ongoing investigation.

### Supplementary Information

**Additional file 1: Figure S1.** Infection downregulates cholesterol biosynthesis genes. **Figure S2. **Influenza infection does not overtly alter myelin proteins but increases marker of OL stress in the mPFC. **Figure S3.** Influenza infection does not alter myelin structure in the mPFC. **Figure S4.** Lipidomics experimental design and statistical analysis. **Figure S5.** Effect of infection on expression levels of Csf1r, Csf1 and Il34 in cerebellum at day 8 p.i.

**Additional file 2: Table S1.** Fold change (FC) and False discovery rate (FDR) of OL-specific, lipid biosynthesis, and myelin-related gene expression.

**Additional file 3: Table S2.** Definitions of measures generated by Imaris.

**Additional file 4. Table S3.** Data output of myelin-related measures of PLP-stained mPFC tissue produced by Imaris Filament Tracer module for PBS- and IAV-inoculated mice at day 8 p.i. and cuprizone-intoxicated mice at 5 weeks post-cuprizone.

**Additional file 5: Table S4.** List of all lipid species of purified mPFC myelin differentially expressed between saline and flu-inoculated mice at day 8 p.i.

**Additional file 6: Table S5.** List of all lipid species of purified mPFC myelin differentially expressed between saline and flu-inoculated mice at day 16 p.i.

**Additional file 7: Table S6.** List of all lipid species of purified mPFC myelin differentially expressed between flu-inoculated mice at day 8 and 16 p.i

**Additional file 8: Table S7.** List of all lipid species of purified whole brain myelin differentially expressed between saline and flu-inoculated mice at day 8 p.i

## Data Availability

All data generated and/or analyzed during the current study are available on request by the corresponding author. The bulk RNA-sequencing dataset associated with this has been deposited into the Gene Expression Omnibus database (https://www.ncbi.nlm.nih.gov/geo/) and can be accessed using the accession no. GSE96870.

## Abbreviations

AcCa: Acyl carnitine
BisMePA: Bismethyl phosphatidic acid
Cer: Ceramide
Cnp: 2′,3′ Cyclic nucleotide phosphodiesterase
dMePE: Dimethyl phosphatidyl ethanolamine
CerG1, CerG2, CerG3: Glucosylceramide or galactosylceramide
CL: Cardiolipin
ChE: Cholesterol
DG: Diglyceride
FA: Fatty acid
LPA: Lysophosphatidic acid
LPC: Lysophosphatidic choline
LPE: Lysophosphatidyl ethanolamine
LPG: Lysophosphatidyl glycerol
LPS: Lysophosphatidic serine
Mag: Myelin-associated glycoprotein
Mbp: Myelin basic protein
MePC: Monoether phosphatidyl choline
MGDG: Monogalactosyldiacylglycerol
MG: Monoglyceride
Mog: Myelin oligodendrocyte glycoprotein
PA: Phosphatidic acid
PC: Phosphatidyl choline
PE: Phosphatidyl ethanolamine
Pet: Phosphatidyl ethanol
PG: Phosphatidyl glycerol
PI: Phosphatidyl inositol
PI(3,4,5)P3: Phosphatidylinositol (3,4,5)-trisphosphate
PLP: Proteolipid protein
PMe: Monomethyl phosphatidyl ethanolamine
PS: Phosphatidyl serine
SM: Sphingomyelin
So: Sphingosine
TG: Triacylglyceride
Ugt8a: UDP galactosyltransferase 8A
