# Supplementary material for: Influenza A virus infection disrupts oligodendrocyte homeostasis and alters the myelin lipidome in the adult mouse
Source: J Neuroinflammation. 2023 Aug 19;20:190. doi: 10.1186/s12974-023-02862-2 (PMC10439573; doi:10.1186/s12974-023-02862-2)
Supplement: Supplementary file 1 — Additional file 1: Figure S1. Infection downregulates cholesterol biosynthesis genes. Figure S2. Influenza infection does not overtly alter myelin proteins but increases marker of OL stress in the mPFC. Figure S3. Influenza infection does not alter myelin structure in the mPFC. Figure S4. Lipidomics experimental design and statistical analysis. Figure S5. Effect of infection on expression levels of Csf1r, Csf1 and Il34 in cerebellum at day 8 p.i. [file 12974_2023_2862_MOESM1_ESM.docx]

**Additional file 1**

**Influenza A virus infection disrupts oligodendrocyte homeostasis and alters the myelin lipidome in the adult mouse.**

^1^Allison Y. Louie^a^, ^1^Justin S. Kim^b,c,d^, Jenny Drnevich^e^, Payam Dibaeinia^f^, Hisami Koito^g^, Saurabh Sinha^b,f,h^, Daniel B. McKim^a,b,j^, Katiria Soto-Diaz^a^, Romana A. Nowak^f,i^, *Aditi Das^a,b,c,d,h^, *Andrew J. Steelman^a,b,h,i^

^1^Equal contribution

*Corresponding authors

Andrew J. Steelman

Email: asteelma@illinois.edu

Aditi Das

Email: aditidas@illinois.edu

**This PDF file includes:**

Figures S1-5

**Figure S1**


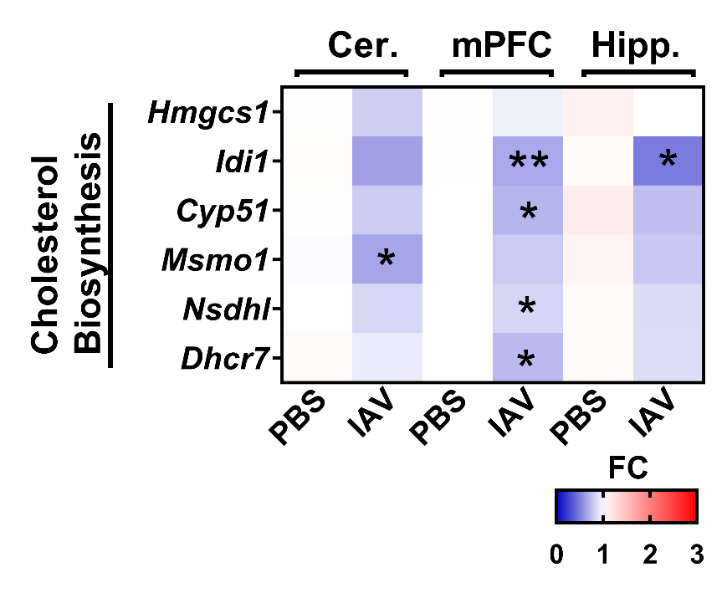


**Figure S1. Infection downregulates cholesterol biosynthesis genes.** Expression of genes involved in cholesterol biosynthesis in the cerebellum (Cer.), medial prefrontal cortex (mPFC), and hippocampus (Hipp.) of IAV-infected mice and PBS controls at day 8 p.i. Shown as fold change (FC) over control. (n=3-5 animals per group). Data analyzed by two-tailed Student’s t test. P-value *<0.05, **<0.01.

**Figure S2**


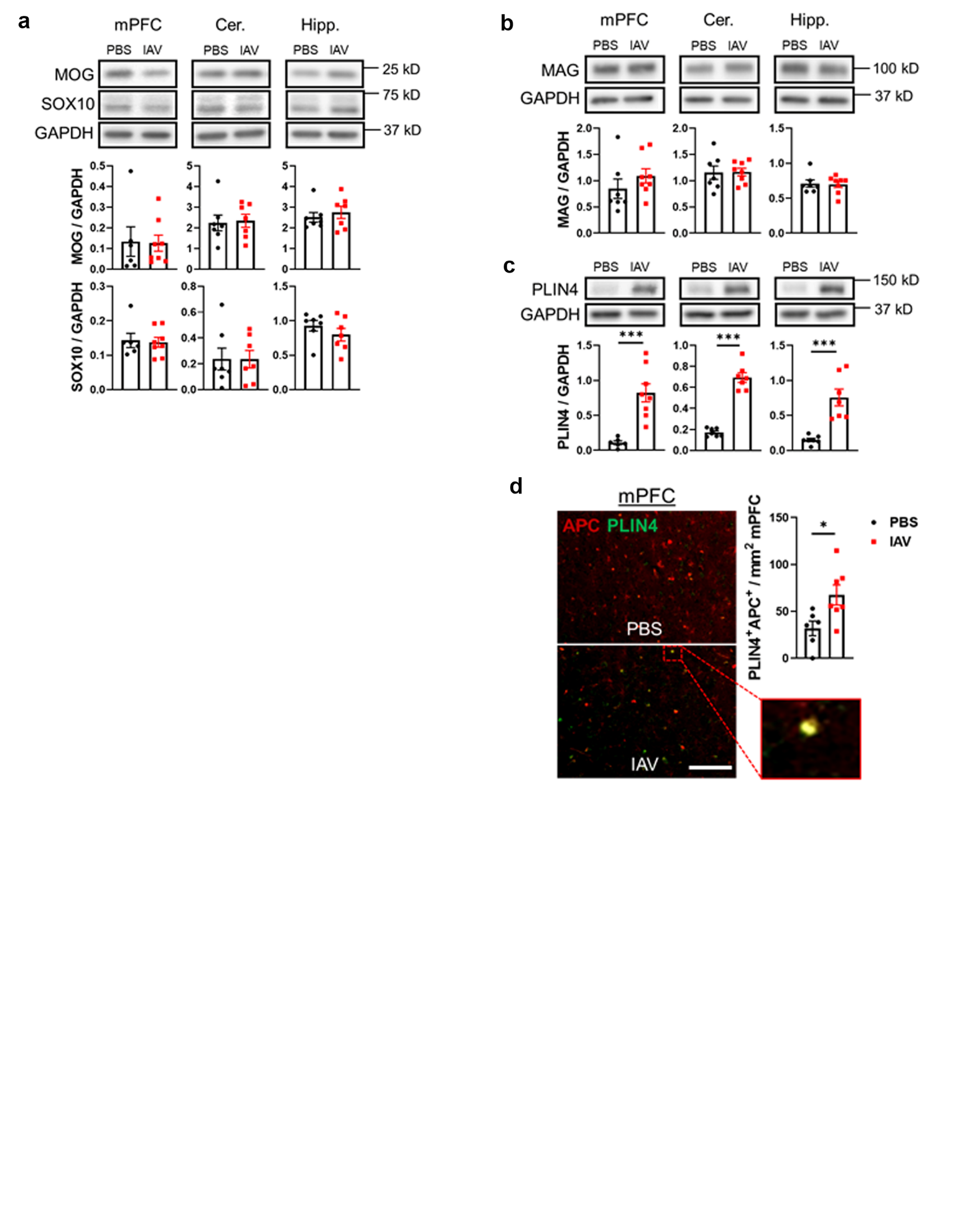


**Figure S2. Influenza infection does not overtly alter myelin proteins but increases marker of OL stress in the mPFC.** (a-b) Immunoblotting analysis with representative blots of OL-specific proteins MOG, SOX10, and MAG in the medial prefrontal cortex (mPFC), cerebellum (Cer.), and hippocampus (Hipp.) of PBS- and IAV-inoculated mice at day 8 p.i. (n=7-8). (c) Immunoblotting analysis with representative blots of OL-stress marker PLIN4 in the mPFC, Cer., and Hipp. of PBS- and IAV-inoculated mice at day 8 p.i. (n=7-8). (d) Representative immunohistochemical staining of mPFC tissue of PBS- and IAV-inoculated mice with anti-APC (CC1 clone) and PLIN4. Number of PLIN4^+^ APC^+^ cells per mm^2^ mPFC (n=6-7) of PBS-and IAV-inoculated mice at day 8 p.i. Data analyzed by Student’s t test and presented as mean ± SEM. P-value *<0.05, ***<0.001.

**Figure S3**


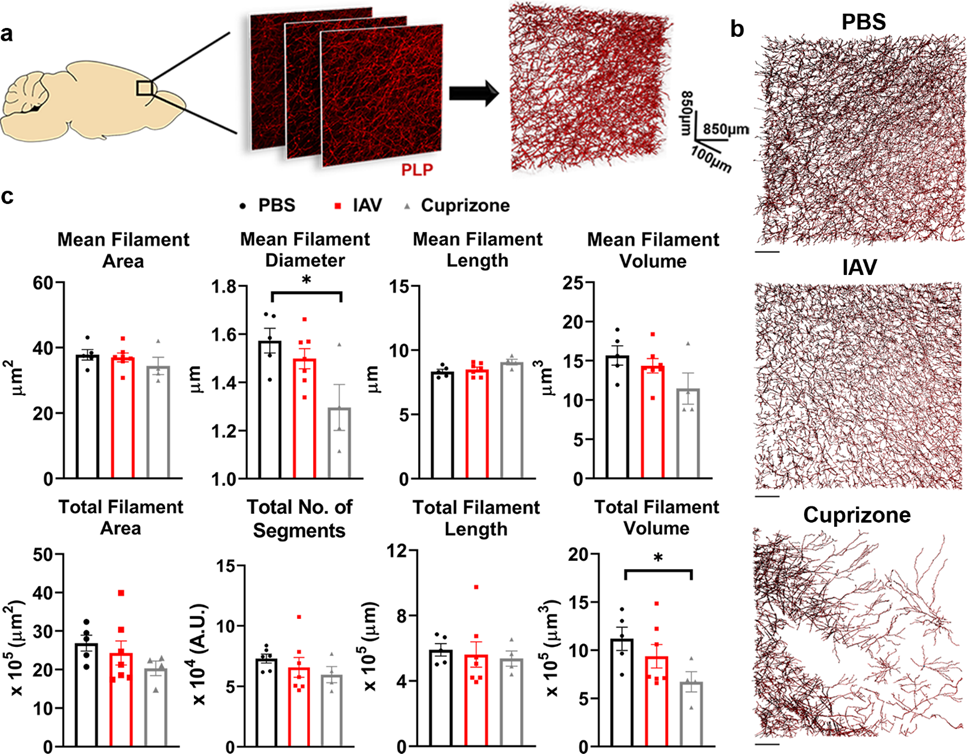


**Figure S3. Influenza infection does not alter myelin structure in the mPFC.** (a) Schematic of CLARITY/immunofluorescence technique to evaluate PLP in the medial prefrontal cortex (mPFC) at day 8 p.i. Dimension bar not drawn to scale. (b) Representative 3D renderings of PLP-stained mPFC tissue of PBS- and IAV-inoculated mice at day 8 p.i., and cuprizone-intoxicated mice 5 wk post-cuprizone. Scale bar is 100 µm. (c) Select measures from Imaris analysis of 3D renderings of PLP-stained mPFC tissue of PBS, IAV, and cuprizone treatment groups at day 8 p.i. (n=4-7, Kruskal-Wallis one-way ANOVA).

**Figure S4**

**
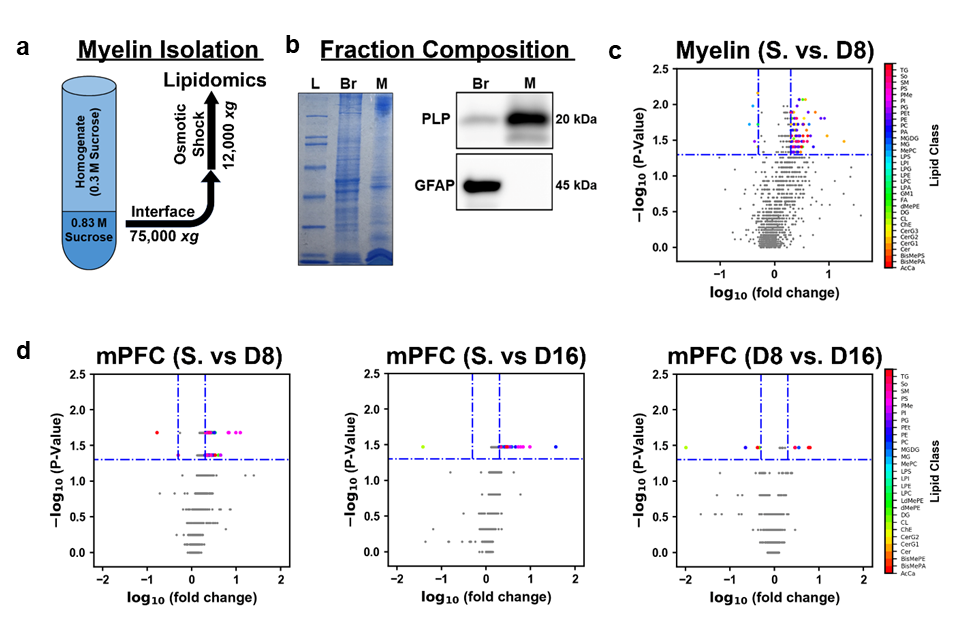
**

**Figure S4. Lipidomics experimental design and statistical analysis.** (a) Experimental design for conducting lipidomics on myelin from PBS- and IAV-inoculated mice at day 8 p.i. (b) Purified myelin isolated via fractionation by ultracentrifugation and osmotic shock was confirmed by SDS PAGE (left) and western blot (right). The fraction composition is denoted by L for ladder, Br for brain fraction, and M for purified myelin. Western blots for proteolipid protein (PLP) found in myelin or glial fibrillary acidic protein (GFAP) found in tissues other than myelin were conducted. (c) Two-sided non-parametric Wilcoxon Ranksum Analysis plotted against fold change of lipid classes from purified myelin of PBS-inoculated (S.) vs. IAV-inoculated mice at day 8 p.i. (D8). Sample size for myelin is n=12 PBS-inoculated mice and n=11 IAV-inoculated mice. (d) Two-sided non-parametric Wilcoxon Ranksum Analysis plotted against fold change of lipid classes from mPFC of PBS-inoculated (S.) vs. IAV-inoculated mice at day 8 p.i. (D8) or day 16 p.i. (D16), and between D8 and D16 (2-3 animals pooled per sample, for n=4 PBS, n=4 IAV D8, and n=3 D16).

**
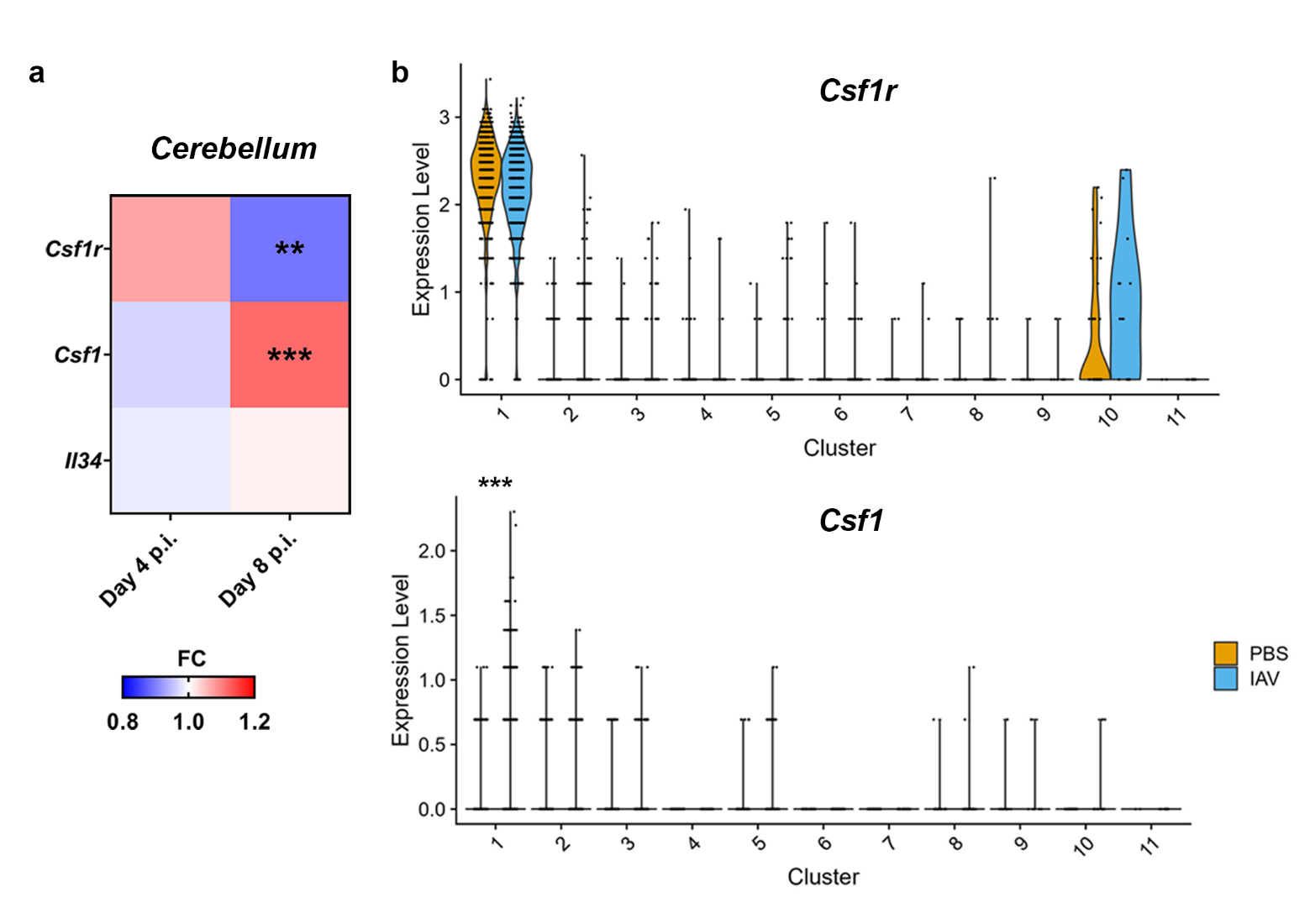
**

**Figure S5**

**Figure S5. Effect of infection on expression levels of *Csf1r*, *Csf1* and *Il34* in cerebellum at day 8 p.i.** Effect of IAV infection on expression of *Csfr1* and its ligands, *Csf1* and *Il34* as determined by bulk RNA-seq. q-value significance **<0.01, ***<0.001. For (a), fold change (FC) shown on log2 scale.
