# Supplementary material for: Influenza A virus infection disrupts oligodendrocyte homeostasis and alters the myelin lipidome in the adult mouse
Source: J Neuroinflammation. 2023 Aug 19;20:190. doi: 10.1186/s12974-023-02862-2 (PMC10439573; doi:10.1186/s12974-023-02862-2)
Supplement: Supplementary file 5 — Additional file 5: Table S4. List of all lipid species of purified mPFC myelin differentially expressed between saline and flu-inoculated mice at day 8 p.i. [file 12974_2023_2862_MOESM5_ESM.pdf]

Table S4. List of all lipid species of purified mPFC myelin differentially expressed between saline and flu-inoculated mice at day 8 p.i.

mPFC (Saline vs. Flu day 8)				
Lipid Ion	***Lipid Class***	***Fold Change (Flu D8/Saline)***	***p-value***	***Representative Structure***
BisMePA(18:2p/14:0)+NH4	BisMePA	2.52	0.04331	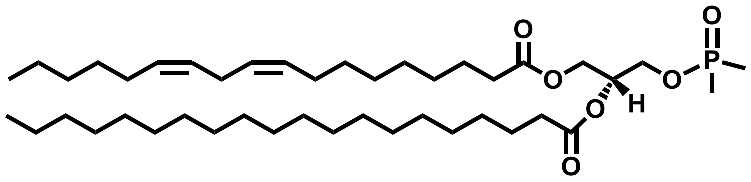 BisMePA (18:2/20:0)
BisMePA(18:2p/20:1)+NH4	BisMePA	1.73	0.04331	
BisMePA(18:2p/22:6)+NH4	BisMePA	1.71	0.04331	
BisMePA(42:10)+NH4	BisMePA	2.21	0.02092	
BisMePA(42:11)+NH4	BisMePA	2.13	0.02092	
BisMePE(16:0/16:0)+H	BisMePE	1.86	0.02092	
BisMePE(18:0/22:6)+H	BisMePE	2.25	0.02092	
Cer(d18:0/24:1)+H	Cer	2.02	0.04331	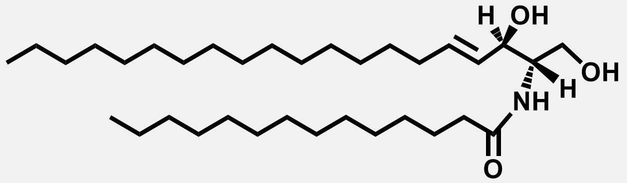 Cer (18:1/14:0)
Cer(d18:1/22:1)+H	Cer	1.61	0.04331	
Cer(d18:1/23:0)+H	Cer	1.55	0.04331	
Cer(d18:1/24:0)+H	Cer	1.88	0.04331	
Cer(d18:1/24:1)+H	Cer	1.71	0.04331	
Cer(d18:2/18:0)+H	Cer	2.70	0.02092	
Cer(d18:2/20:0)+H	Cer	2.43	0.02092	
Cer(d18:2/22:0)+H	Cer	2.22	0.04331	
Cer(d18:2/23:0)+H	Cer	2.26	0.04331	
CerG1(d18:1/20:0)+H	CerG1	1.81	0.04331	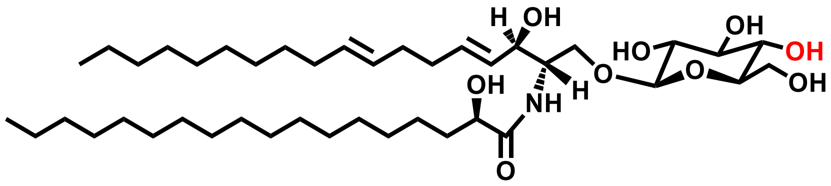 Glucosylceramide (18:2/18:0)
CerG1(d18:1/22:1)+H	CerG1	1.60	0.04331	
CerG1(d18:1/24:0)+H	CerG1	1.95	0.04331	
CerG1(d18:1/24:1)+H	CerG1	1.59	0.04331	
CerG1(d18:1/24:3)+H	CerG1	1.61	0.04331	
CerG1(d18:2/18:0)+H	CerG1	2.83	0.04331	
CerG1(d18:2/25:0)+H	CerG1	2.25	0.04331	
CerG1(d22:1/24:1)+H	CerG1	2.02	0.04331	
CerG1(d36:2)+H	CerG1	1.78	0.04331	
CerG1(d41:6)+H	CerG1	2.07	0.02092	
CerG1(d42:4)+H	CerG1	1.92	0.04331	
CerG2(d18:1/24:1)+H	CerG2	1.63	0.04331	

CerG2(d54:5)+H	CerG2	2.28	0.04331	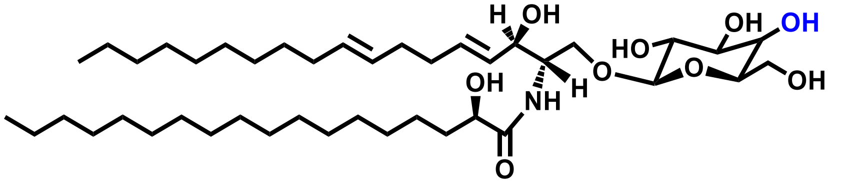 Galactosylceramide (18:2/18:0)
CL(17:1/18:1/18:1/18:2)-H	CL	0.80	0.02092	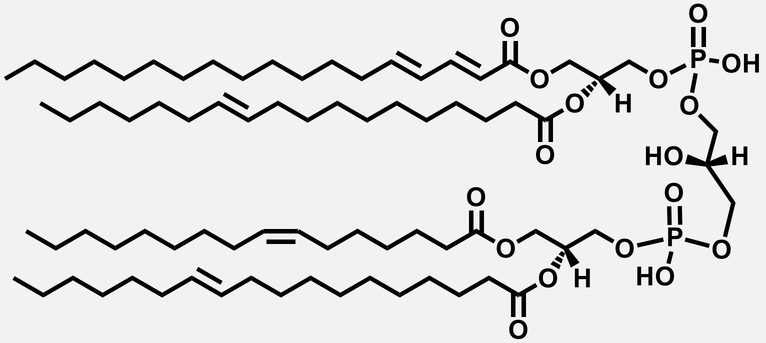 CL (18:2/18:1/16:1/18:1)
CL(18:1/16:1/16:0/22:6)-H	CL	2.90	0.02092	
CL(18:1/18:0/20:3/20:3)-H	CL	1.66	0.02092	
CL(18:1/18:1/18:1/22:6)-H	CL	2.20	0.02092	
CL(18:2/16:0/18:1/20:4)-H	CL	1.44	0.02092	
CL(18:2/16:1/18:2/18:1)-H	CL	1.29	0.02092	
CL(18:2/18:0/20:3/18:1)-H	CL	1.53	0.02092	
CL(18:2/18:1/16:1/18:1)-H	CL	1.43	0.04331	
CL(18:2/18:1/18:1/18:1)-H	CL	1.34	0.02092	
CL(18:2/18:1/18:1/18:2)-H	CL	1.71	0.02092	
CL(18:2/18:1/18:1/20:4)-H	CL	1.68	0.02092	
CL(18:2/20:4/16:1/20:4)-H	CL	1.60	0.02092	
CL(18:2/20:4/18:1/20:4)-H	CL	1.54	0.02092	
CL(18:2/20:4/18:2/20:4)-H	CL	1.61	0.02092	
CL(18:2/20:4/20:4/20:4)-H	CL	1.50	0.02092	
CL(18:2/20:4/22:6/20:4)-H	CL	1.13	0.02092	
CL(18:2/20:4/22:6/22:6)-H	CL	1.83	0.02092	
CL(18:2/22:6/20:4/22:6)-H	CL	1.70	0.02092	
CL(18:4/20:4/16:1/20:4)-H	CL	1.52	0.04331	
CL(18:4/20:4/22:6/20:4)-H	CL	1.88	0.04331	
CL(20:0/18:0/18:0/18:0)-H	CL	1.43	0.02092	
CL(20:2/18:1/18:1/22:6)-H	CL	1.64	0.02092	
CL(20:2/18:1/20:4/20:4)-H	CL	2.14	0.02092	
CL(20:4/16:0/18:1/18:1)-H	CL	2.12	0.04331	
CL(20:4/16:1/16:1/20:4)-H	CL	1.62	0.04331	
CL(20:4/16:1/16:1/22:6)-H	CL	1.86	0.04331	
CL(20:4/16:1/18:1/20:4)-H	CL	1.69	0.02092	
CL(20:4/18:1/16:1/18:1)-H	CL	1.50	0.02092	

CL(20:4/18:1/18:1/18:1)-H	CL	1.49	0.04331	
CL(20:4/18:1/18:1/20:4)-H	CL	2.19	0.02092	
CL(20:4/18:1/20:4/20:4)-H	CL	1.65	0.02092	
CL(20:4/20:4/16:1/20:4)-H	CL	1.47	0.02092	
CL(20:4/20:4/22:6/18:2)-H	CL	2.12	0.02092	
CL(20:5/16:1/22:6/18:2)-H	CL	1.57	0.02092	
CL(20:5/18:0/18:1/20:4)-H	CL	1.74	0.04331	
CL(20:5/20:4/18:2/20:4)-H	CL	1.83	0.02092	
CL(20:5/22:6/18:2/22:6)-H	CL	1.52	0.02092	
CL(21:0/16:0/18:1/22:6)-H	CL	1.49	0.02092	
CL(21:0/16:0/20:3/22:6)-H	CL	1.56	0.04331	
CL(21:0/22:6/16:0/18:1)-H	CL	2.49	0.02092	
CL(22:0/16:0/18:0/18:1)-H	CL	1.64	0.04331	
CL(22:1/18:0/18:0/18:0)-H	CL	1.59	0.02092	
CL(22:4/18:1/18:1/18:2)-H	CL	1.74	0.02092	
CL(22:6/16:0/16:0/16:0)-H	CL	2.04	0.04331	
CL(22:6/16:1/16:1/22:6)-H	CL	1.66	0.04331	
CL(22:6/18:0/20:0/22:4)-H	CL	1.64	0.02092	
CL(22:6/18:0/20:4/22:1)-H	CL	1.55	0.02092	
CL(22:6/18:1/16:1/18:1)-H	CL	1.65	0.02092	
CL(22:6/18:1/20:4/22:6)-H	CL	1.75	0.02092	
CL(22:6/20:4/16:1/20:4)-H	CL	1.91	0.04331	
CL(22:6/20:4/20:4/22:6)-H	CL	2.02	0.02092	
CL(22:6/22:6/22:6/18:2)-H	CL	1.71	0.02092	
CL(23:0/16:0/16:0/18:1)-H	CL	1.37	0.02092	
CL(23:0/16:0/20:4/22:6)-H	CL	1.60	0.02092	
CL(24:2/16:0/20:4/18:0)-H	CL	1.51	0.04331	
CL(77:4)-H	CL	1.47	0.04331	
CL(82:3)-H	CL	1.32	0.02092	
DG(16:0/20:4)+NH4	DG	1.78	0.02092	
DG(16:0/22:6)+NH4	DG	2.60	0.04331	
DG(18:0/18:1)+NH4	DG	1.24	0.04331	
DG(18:0/18:2)+NH4	DG	1.92	0.02092	
DG(18:0/20:1)+NH4	DG	1.21	0.04331	

DG(18:0/20:2)+NH4	DG	2.18	0.04331	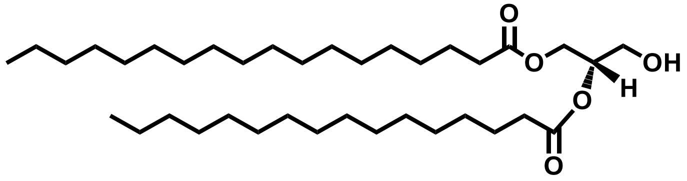 DG (16:0/18:0)
DG(18:0/20:3)+NH4	DG	2.54	0.04331	
DG(18:0/20:4)+NH4	DG	2.08	0.02092	
DG(18:0/22:1)+NH4	DG	1.32	0.04331	
DG(18:0/22:4)+NH4	DG	2.42	0.04331	
DG(18:0/22:6)+NH4	DG	2.11	0.02092	
DG(18:1/18:1)+NH4	DG	2.67	0.02092	
DG(18:1/20:4)+NH4	DG	2.27	0.02092	
DG(18:1/22:1)+NH4	DG	3.99	0.04331	
DG(18:1/22:6)+NH4	DG	2.36	0.02092	
DG(18:1/23:0)+NH4	DG	2.41	0.02092	
DG(18:1/24:0)+NH4	DG	3.06	0.04331	
DG(18:1/24:1)+NH4	DG	1.68	0.02092	
DG(20:1/18:1)+NH4	DG	2.24	0.04331	
dMePE(16:0/18:1)-H	dMePE	1.34	0.04331	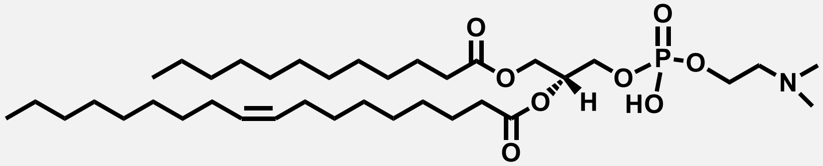 dMePE (16:0/18:1)
dMePE(16:0/22:6)-H	dMePE	1.72	0.02092	
dMePE(16:0p/24:1)-H	dMePE	1.27	0.04331	
dMePE(16:1p/24:1)-H	dMePE	1.94	0.04331	
dMePE(18:0/18:1)-H	dMePE	1.31	0.04331	
dMePE(18:0/22:5)-H	dMePE	1.43	0.02092	
dMePE(18:2p/20:4)-H	dMePE	1.31	0.04331	
dMePE(22:6/22:6)-H	dMePE	1.75	0.02092	
dMePE(38:0p)-H	dMePE	1.42	0.04331	
dMePE(38:4)-H	dMePE	1.41	0.02092	
dMePE(38:7)-H	dMePE	1.84	0.02092	
dMePE(54:5)-H	dMePE	1.74	0.04331	
LdMePE(18:1)-H	LdMePE	1.52	0.02092	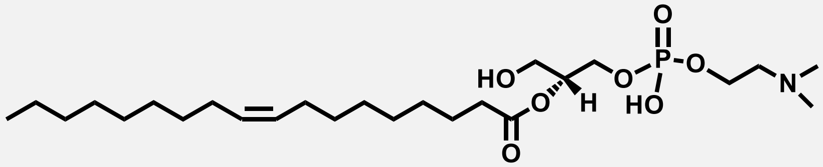 LdMePE (16:0/18:1)
LPC(12:0)+H	LPC	1.83	0.02092	
LPC(16:0e)+H	LPC	1.42	0.02092	

LPC(18:0)+H	LPC	1.77	0.04331	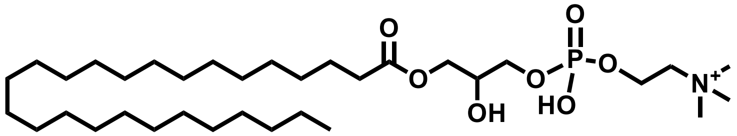 LPC (26:0)
LPC(18:0e)+H	LPC	1.80	0.04331	
LPC(18:0p)+H	LPC	1.78	0.02092	
LPC(18:2)+H	LPC	1.80	0.04331	
LPC(22:0)+H	LPC	1.31	0.04331	
LPC(23:0)+H	LPC	1.55	0.02092	
LPC(24:0)+H	LPC	2.65	0.02092	
LPC(24:1)+H	LPC	2.77	0.02092	
LPE(16:0)-H	LPE	0.69	0.02092	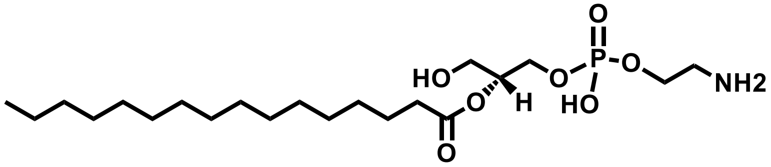 LPE (16:0)
MePC(30:0)+NH4	MePC	1.99	0.02092	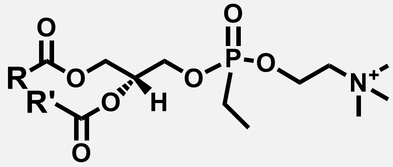 MePC
MePC(32:3)+NH4	MePC	1.46	0.04331	
MePC(53:3)+NH4	MePC	1.79	0.04331	
MG(18:0)+H	MG	1.29	0.04331	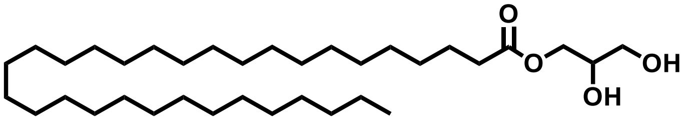 MG (32:0)
MG(32:0)+H	MG	1.35	0.04331	
MGDG(37:5)-H	MGDG	1.07	0.04331	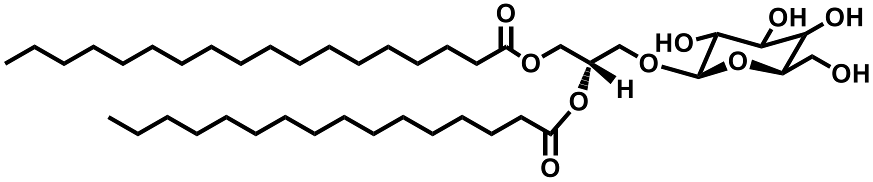 MGDG (16:0/18:0)
MGDG(39:5)-H	MGDG	1.48	0.02092	
MGDG(44:10)-H	MGDG	1.41	0.02092	
MGDG(46:4)-H	MGDG	1.38	0.02092	
PC(16:0p/22:6)+H	PC	2.23	0.04331	
PC(17:1/16:1)+H	PC	1.53	0.04331	

PC(18:1/22:6)+H	PC	1.99	0.04331	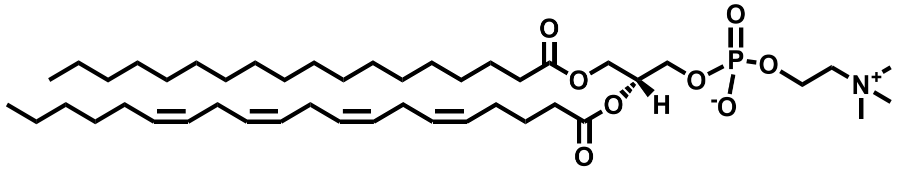 PC (18:0/20:4)
PC(18:1p/16:0)+H	PC	1.41	0.02092	
PC(20:1p/16:0)+H	PC	1.44	0.02092	
PC(23:0e)+H	PC	1.01	0.04331	
PC(31:3)+H	PC	1.33	0.04331	
PC(33:3)+H	PC	1.27	0.04331	
PC(34:2)+H	PC	1.98	0.04331	
PC(34:3)+H	PC	1.87	0.02092	
PC(34:3p)+H	PC	2.47	0.04331	
PC(36:4e)+H	PC	2.01	0.02092	
PC(36:5p)+H	PC	1.44	0.02092	
PC(38:4p)+H	PC	1.46	0.02092	
PC(38:6e)+H	PC	1.50	0.02092	
PC(40:6p)+H	PC	1.64	0.02092	
PC(40:8)+H	PC	1.51	0.04331	
PC(42:11)+H	PC	1.78	0.04331	
PC(46:5)+H	PC	2.58	0.04331	
PE(16:0/16:1)+H	PE	0.95	0.04331	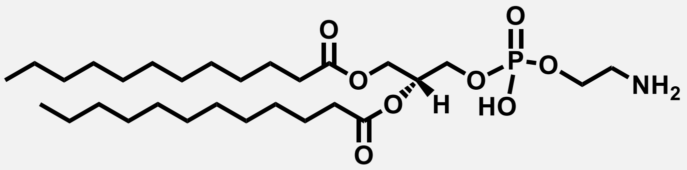 PE (12:0/12:0)
PE(18:0/18:2)+H	PE	1.61	0.04331	
PE(18:1/18:2)+H	PE	1.69	0.04331	
PE(18:1/18:3)+H	PE	1.42	0.02092	
PE(18:1/22:6)+H	PE	1.77	0.04331	
PE(18:1p/20:1)+H	PE	1.70	0.04331	
PE(18:2/20:4)+H	PE	1.27	0.04331	
PE(18:2/22:6)+H	PE	1.24	0.02092	
PE(18:2p/22:6)+H	PE	2.29	0.02092	
PE(18:3/22:6)+H	PE	2.06	0.04331	
PE(20:4e)+H	PE	1.17	0.04331	
PE(22:4/20:4)+H	PE	1.29	0.04331	
PE(38:4p)+H	PE	1.36	0.02092	
PE(38:5)+H	PE	1.42	0.02092	
PE(38:6e)+H	PE	2.27	0.04331	
PE(40:7)+H	PE	1.49	0.02092	
PG(12:0/14:0)-H	PG	1.54	0.04331	

PG(16:0/18:1)-H	PG	1.61	0.04331	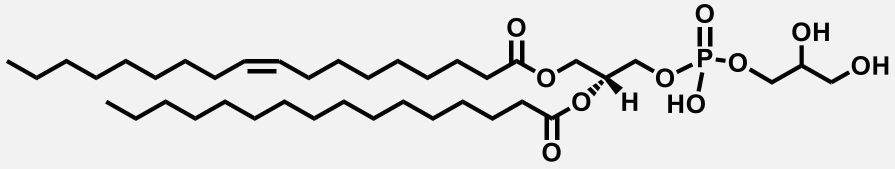 PG (18:1/16:0)
PG(16:0/18:2)-H	PG	1.63	0.04331	
PG(17:0/17:0)-H	PG	1.10	0.02092	
PG(18:1/18:2)-H	PG	1.41	0.02092	
PG(18:2/20:4)-H	PG	1.16	0.02092	
PI(16:0/18:1)-H	PI	1.52	0.04331	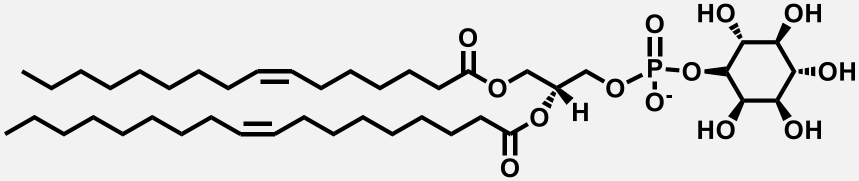 PI (16:1/18:0)
PI(16:0/20:5)-H	PI	1.20	0.04331	
PI(16:1/18:1)-H	PI	1.52	0.02092	
PI(16:1/20:4)-H	PI	4.47	0.04331	
PI(18:1/20:4)-H	PI	1.96	0.02092	
PI(18:1/22:6)-H	PI	1.50	0.04331	
PI(18:1p/20:4)-H	PI	1.62	0.02092	
PI(18:2/20:4)-H	PI	1.62	0.02092	
PI(20:4/20:4)-H	PI	1.81	0.02092	
PI(20:4/22:6)-H	PI	2.30	0.02092	
PI(37:1)-H	PI	2.21	0.02092	
PI(41:6)-H	PI	2.45	0.02092	
PMe(16:0/16:1)-H	PMe	2.20	0.02092	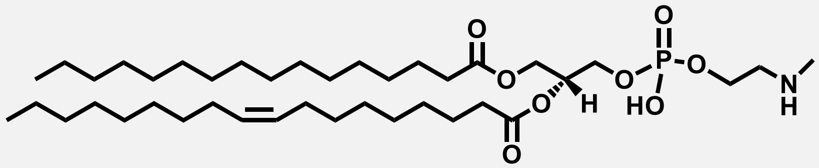 PMe (16:0/18:1)
PMe(16:0/18:1)-H	PMe	6.87	0.02092	
PMe(16:0/20:4)-H	PMe	9.83	0.02092	
PMe(18:0/18:1)-H	PMe	12.35	0.02092	
PS(12:0/14:0)-H	PS	1.59	0.02092	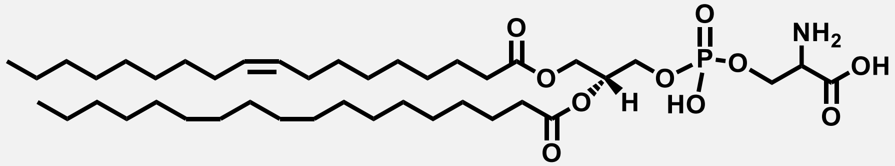 PS (18:1/18:2)
PS(18:1/24:1)-H	PS	2.13	0.04331	
PS(20:1/18:1)-H	PS	1.34	0.02092	
PS(20:4/20:4)-H	PS	1.51	0.02092	
PS(22:6/22:6)-H	PS	1.63	0.04331	
PS(24:0/20:4)-H	PS	2.12	0.04331	
PS(37:2)-H	PS	1.37	0.04331	
PS(38:3p)-H	PS	1.55	0.04331	
PS(39:3)-H	PS	1.34	0.02092	
PS(41:5)-H	PS	1.35	0.02092	

PS(41:6)-H	PS	2.64	0.04331	
PS(45:1)-H	PS	1.43	0.04331	
SM(d18:1/22:1)+H	SM	1.49	0.02092	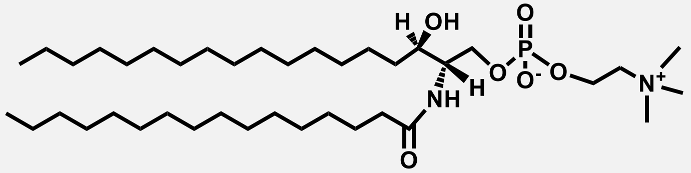 **SM (d18:0/16:0)**
SM(d37:1)+H	SM	1.68	0.04331	
SM(d38:1)+H	SM	1.36	0.04331	
SM(d40:2)+H	SM	2.48	0.04331	
So(d18:0)+H	So	1.62	0.02092	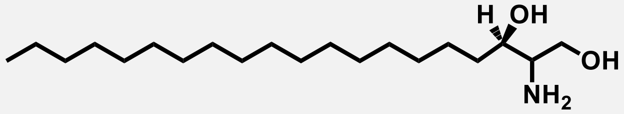 **So (18:0)**
TG(16:1/16:1/18:2)+NH4	TG	1.16	0.02092	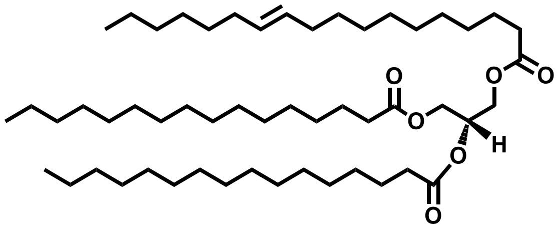 **TG (16:0/16:0/18:1(11E))**
TG(68:4)+NH4	TG	1.61	0.04331	
TG(8:0/8:0/8:0)+NH4	TG	2.11	0.04331	
