# Supplementary material for: Influenza A virus infection disrupts oligodendrocyte homeostasis and alters the myelin lipidome in the adult mouse
Source: J Neuroinflammation. 2023 Aug 19;20:190. doi: 10.1186/s12974-023-02862-2 (PMC10439573; doi:10.1186/s12974-023-02862-2)
Supplement: Supplementary file 6 — Additional file 6: Table S5. List of all lipid species of purified mPFC myelin differentially expressed between saline and flu-inoculated mice at day 16 p.i. [file 12974_2023_2862_MOESM6_ESM.pdf]

Table S5. List of all lipid species of purified mPFC myelin differentially expressed between saline and flu-inoculated mice at day 16 p.i.

mPFC (Saline vs. Flu day 16)				
Lipid Ion	***Lipid Class***	***Fold Change (Flu D16/Saline)***	***p-value***	***Representative Structures***
AcCa(12:0)+H	AcCa	1.99	0.03389	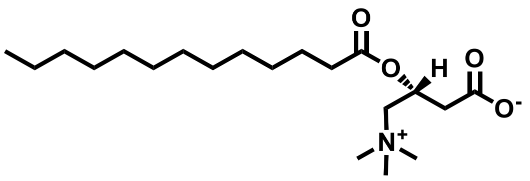 **AcCa (13:0)**
AcCa(14:0)+H	AcCa	1.92	0.03389	
AcCa(14:1)+H	AcCa	1.84	0.03389	
AcCa(16:0)+H	AcCa	1.91	0.03389	
AcCa(16:1)+H	AcCa	1.86	0.03389	
AcCa(18:0)+H	AcCa	1.88	0.03389	
AcCa(18:1)+H	AcCa	2.22	0.03389	
AcCa(18:2)+H	AcCa	2.26	0.03389	
AcCa(20:1)+H	AcCa	1.62	0.03389	
AcCa(20:4)+H	AcCa	2.27	0.03389	
BisMePA(10:0p/10:4)+NH4	BisMePA	1.68	0.03389	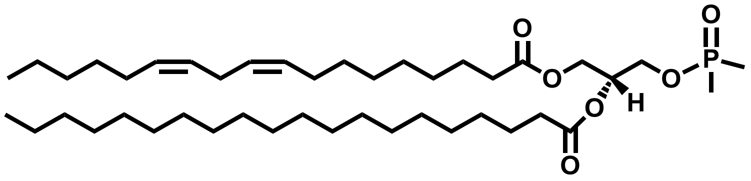 **BisMePA (18:2/20:0)**
BisMePA(16:0/20:5)+NH4	BisMePA	1.54	0.03389	
BisMePA(18:0/20:4)+NH4	BisMePA	1.60	0.03389	
BisMePA(18:0/20:5)+NH4	BisMePA	1.55	0.03389	
BisMePA(18:0/22:5)+NH4	BisMePA	1.39	0.03389	
BisMePA(18:1/20:5)+NH4	BisMePA	1.58	0.03389	
BisMePA(18:2p/16:0)+NH4	BisMePA	1.76	0.03389	
BisMePA(18:2p/18:1)+NH4	BisMePA	1.83	0.03389	
BisMePA(18:2p/20:3)+NH4	BisMePA	1.63	0.03389	
BisMePA(18:2p/22:6)+NH4	BisMePA	1.62	0.03389	
BisMePA(22:5/22:6)+NH4	BisMePA	1.77	0.03389	
BisMePA(22:6/22:6)+NH4	BisMePA	1.66	0.03389	
BisMePA(42:11)+NH4	BisMePA	1.64	0.03389	
BisMePE(18:0/22:6)+H	BisMePE	2.05	0.03389	
Cer(d18:0/12:0)+H	Cer	1.49	0.03389	
Cer(d18:0/24:1)+H	Cer	2.03	0.03389	
Cer(d18:1/22:1)+H	Cer	1.65	0.03389	
Cer(d18:1/24:2)+H	Cer	1.58	0.03389	
Cer(d18:2/18:0)+H	Cer	1.75	0.03389	

Cer(d18:2/20:0)+H	Cer	2.01	0.03389
Cer(d18:2/22:0)+H	Cer	1.73	0.03389
Cer(d18:2/22:1)+H	Cer	1.97	0.03389
Cer(d18:2/23:1)+H	Cer	1.80	0.03389
Cer(d18:2/24:1)+H	Cer	2.05	0.03389
Cer(d18:2/24:2)+H	Cer	2.06	0.03389
CerG1(d18:0/18:0)+H	CerG1	2.23	0.03389
CerG1(d18:0/24:1)+H	CerG1	1.93	0.03389
CerG1(d18:1/16:0)+H	CerG1	1.89	0.03389
CerG1(d18:1/18:0)+H	CerG1	1.69	0.03389
CerG1(d18:1/18:1)+H	CerG1	1.42	0.03389
CerG1(d18:1/22:0)+H	CerG1	1.88	0.03389
CerG1(d18:1/22:1)+H	CerG1	1.64	0.03389
CerG1(d18:1/23:1)+H	CerG1	1.51	0.03389
CerG1(d18:1/24:2)+H	CerG1	1.63	0.03389
CerG1(d18:1/24:3)+H	CerG1	1.90	0.03389
CerG1(d18:1/26:3)+H	CerG1	1.70	0.03389
CerG1(d18:2/18:0)+H	CerG1	2.60	0.03389
CerG1(d18:2/18:1)+H	CerG1	1.79	0.03389
CerG1(d18:2/22:1)+H	CerG1	1.91	0.03389
CerG1(d18:2/23:0)+H	CerG1	2.08	0.03389
CerG1(d18:2/23:1)+H	CerG1	1.86	0.03389
CerG1(d18:2/24:0)+H	CerG1	2.15	0.03389
CerG1(d18:2/24:1)+H	CerG1	2.10	0.03389
CerG1(d36:2)+H	CerG1	2.55	0.03389
CerG1(d39:4)+H	CerG1	1.60	0.03389
CerG1(d40:3)+H	CerG1	1.75	0.03389
CerG1(d41:4)+H	CerG1	1.43	0.03389
CerG1(d41:5)+H	CerG1	1.68	0.03389
CerG1(d41:6)+H	CerG1	1.81	0.03389
CerG1(d42:4)+H	CerG1	1.79	0.03389
CerG1(d42:8)+H	CerG1	1.42	0.03389
CerG1(d44:5)+H	CerG1	1.66	0.03389

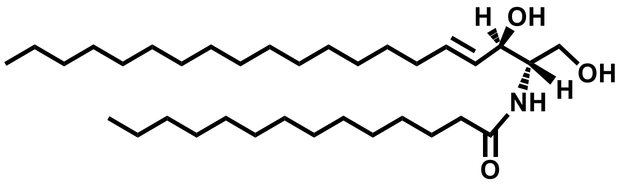

Cer (18:1/14:0)

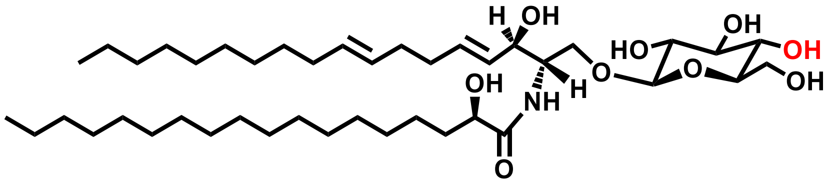

Glucosylceramide (18:2/18:0)

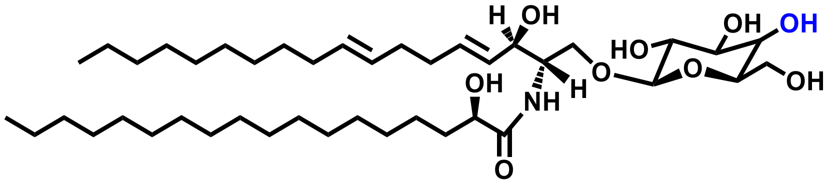

Galactosylceramide (18:2/18:0)

ChE(24:6)+NH4	ChE	0.07	0.03389	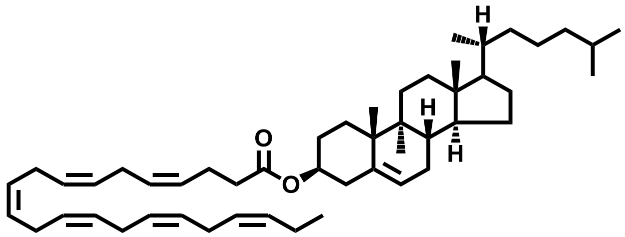 ChE (22:6)
CL(17:1/18:2/20:4/18:1)-H	CL	1.36	0.03389	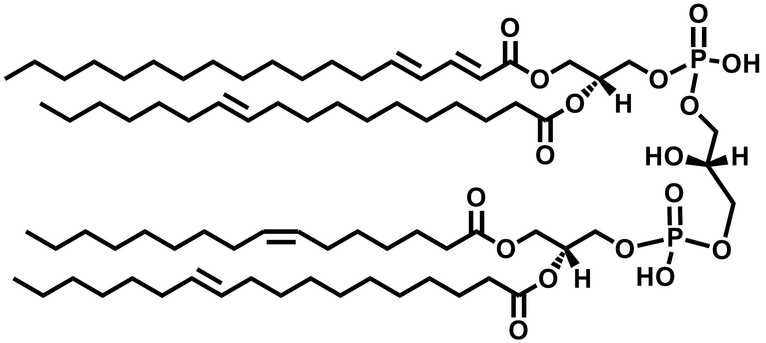 CL (18:2/18:1/16:1/18:1)
CL(18:1/16:1/16:0/22:6)-H	CL	1.34	0.03389	
CL(18:1/18:1/18:1/22:6)-H	CL	1.70	0.03389	
CL(18:1/20:3/20:4/20:4)-H	CL	1.94	0.03389	
CL(18:2/14:0/14:0/14:0)-H	CL	0.04	0.03389	
CL(18:2/16:0/18:1/20:4)-H	CL	1.29	0.03389	
CL(18:2/20:4/18:2/20:4)-H	CL	1.49	0.03389	
CL(18:2/20:4/22:6/18:2)-H	CL	1.55	0.03389	
CL(18:4/22:6/20:4/22:6)-H	CL	1.27	0.03389	
CL(20:0/18:0/18:0/18:0)-H	CL	1.75	0.03389	
CL(20:2/18:1/20:4/20:4)-H	CL	1.67	0.03389	
CL(20:4/16:1/16:1/22:6)-H	CL	1.60	0.03389	
CL(20:4/18:1/18:1/20:4)-H	CL	1.89	0.03389	
CL(20:4/18:1/18:1/22:6)-H	CL	1.79	0.03389	
CL(20:4/18:1/20:4/20:4)-H	CL	1.29	0.03389	
CL(20:4/18:1/20:4/22:6)-H	CL	1.39	0.03389	
CL(20:4/20:4/16:1/20:4)-H	CL	1.39	0.03389	
CL(20:4/20:4/22:6/18:2)-H	CL	1.58	0.03389	
CL(22:1/18:0/18:0/18:0)-H	CL	1.64	0.03389	
CL(22:4/18:1/18:1/18:2)-H	CL	1.46	0.03389	
CL(22:6/16:0/20:0/20:4)-H	CL	1.39	0.03389	
CL(22:6/16:0/20:1/20:4)-H	CL	1.56	0.03389	
CL(22:6/20:4/20:4/22:6)-H	CL	1.71	0.03389	
CL(23:0/16:0/20:4/22:6)-H	CL	1.49	0.03389	
CL(75:6)-H	CL	1.01	0.03389	
DG(16:0/18:1)+NH4	DG	1.15	0.03389	

DG(16:0/20:3)+NH4	DG	1.60	0.03389	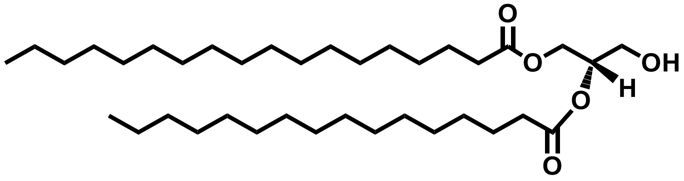
DG(16:0/20:4)+NH4	DG	1.52	0.03389	DG (16:0/18:0)
DG(16:0/22:6)+NH4	DG	1.59	0.03389	
DG(18:0/18:2)+NH4	DG	1.58	0.03389	
DG(18:0/20:3)+NH4	DG	1.96	0.03389	
DG(18:0/20:4)+NH4	DG	1.76	0.03389	
DG(18:0/22:1)+NH4	DG	1.10	0.03389	
DG(18:0/22:4)+NH4	DG	1.94	0.03389	
DG(18:0/22:6)+NH4	DG	2.03	0.03389	
DG(18:1/18:1)+NH4	DG	2.08	0.03389	
DG(18:1/20:4)+NH4	DG	2.27	0.03389	
DG(18:1/22:6)+NH4	DG	1.93	0.03389	
DG(18:1/23:0)+NH4	DG	2.48	0.03389	
DG(18:1/24:0)+NH4	DG	2.12	0.03389	
DG(18:1/24:1)+NH4	DG	2.15	0.03389	
DG(20:1/18:1)+NH4	DG	1.84	0.03389	
DG(20:4/22:6)+NH4	DG	2.29	0.03389	
dMePE(12:0/12:0)-H	dMePE	0.93	0.03389	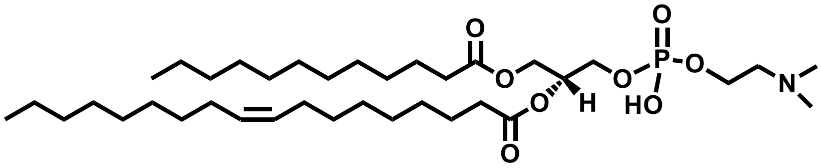
dMePE(16:0/18:1)-H	dMePE	1.52	0.03389	dMePE (16:0/18:1)
dMePE(16:1p/24:1)-H	dMePE	1.60	0.03389	
dMePE(18:0/16:0)-H	dMePE	1.44	0.03389	
dMePE(18:0/20:4)-H	dMePE	2.17	0.03389	
dMePE(20:4/22:6)-H	dMePE	1.50	0.03389	
dMePE(22:6/22:6)-H	dMePE	1.87	0.03389	
dMePE(52:2)-H	dMePE	1.94	0.03389	
dMePE(54:5)-H	dMePE	2.00	0.03389	
LPC(14:0)+H	LPC	1.53	0.03389	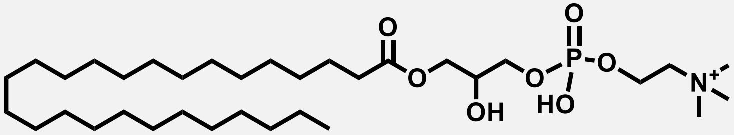
LPC(16:0e)+H	LPC	0.78	0.03389	LPC (26:0)
LPC(16:0p)+H	LPC	2.02	0.03389	
LPC(16:1)+H	LPC	1.70	0.03389	
LPC(18:0p)+H	LPC	1.48	0.03389	
LPC(18:1p)+H	LPC	1.31	0.03389	
LPC(18:3)+H	LPC	1.46	0.03389	
LPC(20:0)+H	LPC	1.51	0.03389	

LPC(20:1)+H	LPC	1.87	0.03389	
LPC(20:4)+H	LPC	1.27	0.03389	
LPC(22:0)+H	LPC	1.61	0.03389	
LPC(22:1)+H	LPC	2.19	0.03389	
LPC(22:6)+H	LPC	1.59	0.03389	
LPC(23:0)+H	LPC	1.88	0.03389	
LPC(24:0)+H	LPC	2.14	0.03389	
LPC(24:1)+H	LPC	2.36	0.03389	
LPE(16:0)-H	LPE	1.12	0.03389	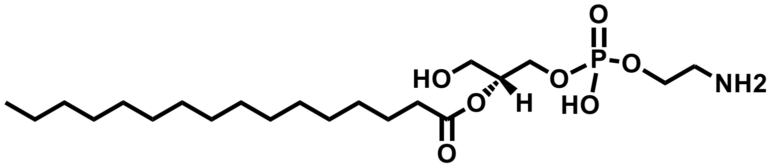 LPE (16:0)
LPE(18:0p)-H	LPE	0.96	0.03389	
LPE(18:1)-H	LPE	1.85	0.03389	
LPE(22:6)-H	LPE	2.21	0.03389	
LPI(18:0)-H	LPI	1.78	0.03389	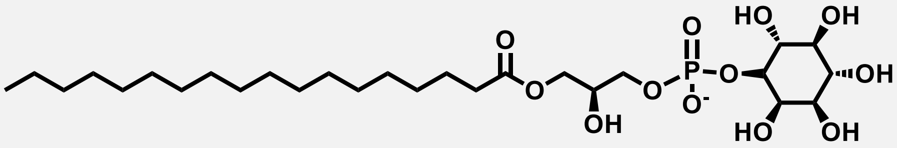 LPI (18:0)
LPS(22:6)-H	LPS	2.05	0.03389	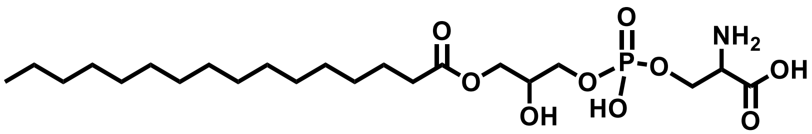 LPS (16:0)
MePC(30:0)+NH4	MePC	1.96	0.03389	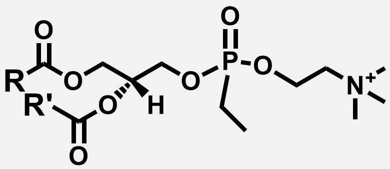 MePC
MePC(32:1)+NH4	MePC	1.76	0.03389	
MePC(33:4)+NH4	MePC	1.63	0.03389	
MePC(34:2)+NH4	MePC	1.57	0.03389	
MePC(38:7)+NH4	MePC	1.58	0.03389	
MePC(38:8)+NH4	MePC	1.46	0.03389	
MePC(51:2)+NH4	MePC	1.46	0.03389	
MePC(51:3)+NH4	MePC	2.37	0.03389	
MePC(53:3)+NH4	MePC	2.01	0.03389	

MG(18:2p)+H	MG	1.92	0.03389	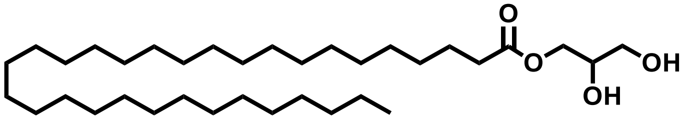 MG (32:0)
MG(32:0)+H	MG	1.74	0.03389	
MG(34:1)+H	MG	1.64	0.03389	
MGDG(15:0/22:4)-H	MGDG	1.75	0.03389	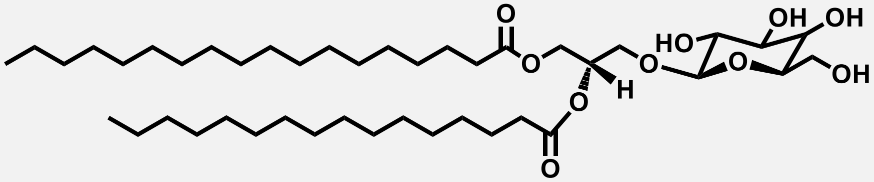 MGDG (16:0/18:0)
MGDG(37:5)-H	MGDG	3.76	0.03389	
MGDG(39:5)-H	MGDG	1.68	0.03389	
MGDG(40:5)-H	MGDG	2.02	0.03389	
MGDG(42:7)-H	MGDG	1.44	0.03389	
MGDG(46:4)-H	MGDG	1.42	0.03389	
PC(15:0/22:6)+H	PC	1.36	0.03389	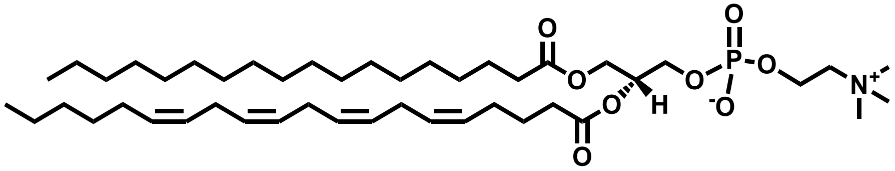 PC (18:0/20:4)
PC(16:0/14:0)+H	PC	1.00	0.03389	
PC(16:0e/22:5)+H	PC	1.34	0.03389	
PC(16:0p/18:1)+H	PC	1.40	0.03389	
PC(16:1/13:0)+H	PC	1.31	0.03389	
PC(17:0/22:6)+H	PC	1.22	0.03389	
PC(18:0/22:4)+H	PC	1.17	0.03389	
PC(18:0p/16:0)+H	PC	1.27	0.03389	
PC(18:0p/18:0)+H	PC	1.10	0.03389	
PC(18:1/13:0)+H	PC	1.19	0.03389	
PC(18:1p/18:0)+H	PC	1.28	0.03389	
PC(19:1/18:1)+H	PC	1.40	0.03389	
PC(20:0/22:6)+H	PC	1.31	0.03389	
PC(20:0p/20:4)+H	PC	1.34	0.03389	
PC(22:0/20:3)+H	PC	1.26	0.03389	
PC(24:0/20:4)+H	PC	0.80	0.03389	
PC(32:4)+H	PC	1.39	0.03389	
PC(33:2)+H	PC	1.24	0.03389	
PC(34:1)+H	PC	1.39	0.03389	

PC(34:2)+H	PC	1.47	0.03389
PC(34:4)+H	PC	0.81	0.03389
PC(36:2)+H	PC	1.37	0.03389
PC(36:4p)+H	PC	1.27	0.03389
PC(37:2)+H	PC	1.32	0.03389
PC(38:2)+H	PC	1.48	0.03389
PC(38:2e)+H	PC	1.71	0.03389
PC(38:4e)+H	PC	1.30	0.03389
PC(38:5)+H	PC	1.53	0.03389
PC(39:7)+H	PC	1.15	0.03389
PC(40:2)+H	PC	1.19	0.03389
PC(40:6)+H	PC	1.10	0.03389
PC(40:6p)+H	PC	1.43	0.03389
PC(40:7p)+H	PC	1.34	0.03389
PC(41:2)+H	PC	1.71	0.03389
PC(41:4)+H	PC	1.32	0.03389
PC(42:2)+H	PC	1.83	0.03389
PC(42:4)+H	PC	1.26	0.03389
PC(43:4)+H	PC	1.60	0.03389
PC(44:1)+H	PC	1.40	0.03389
PC(44:11)+H	PC	1.36	0.03389
PC(44:2)+H	PC	1.33	0.03389
PC(44:4)+H	PC	1.71	0.03389
PC(44:5)+H	PC	1.95	0.03389
PC(44:7)+H	PC	2.19	0.03389
PC(45:5)+H	PC	1.96	0.03389
PC(46:5)+H	PC	1.94	0.03389
PE(10:0e/10:4)+H	PE	1.61	0.03389
PE(12:0/14:0)+H	PE	0.90	0.03389
PE(14:0/20:4)+H	PE	1.23	0.03389
PE(14:0/22:6)+H	PE	0.91	0.03389
PE(16:0/18:1)+H	PE	1.24	0.03389
PE(16:0/20:1)+H	PE	1.30	0.03389
PE(16:0/20:4)+H	PE	1.53	0.03389

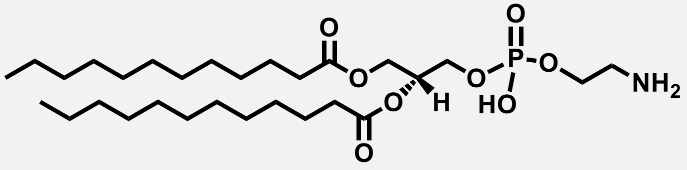

PE (12:0/12:0)

PE(16:0/22:4)+H	PE	1.54	0.03389	
PE(16:0/22:6)+H	PE	1.61	0.03389	
PE(16:0e)+H	PE	1.21	0.03389	
PE(16:0p/18:1)+H	PE	1.49	0.03389	
PE(16:0p/19:1)+H	PE	1.56	0.03389	
PE(16:0p/20:3)+H	PE	1.68	0.03389	
PE(16:0p/22:4)+H	PE	1.48	0.03389	
PE(16:0p/22:5)+H	PE	1.56	0.03389	
PE(16:1/20:4)+H	PE	1.04	0.03389	
PE(16:1/22:6)+H	PE	1.52	0.03389	
PE(17:0/18:1)+H	PE	1.31	0.03389	
PE(17:1/22:6)+H	PE	1.65	0.03389	
PE(18:0/18:1)+H	PE	1.80	0.03389	
PE(18:0/18:2)+H	PE	1.62	0.03389	
PE(18:0/20:4)+H	PE	1.40	0.03389	
PE(18:0/22:4)+H	PE	1.37	0.03389	
PE(18:0/22:6)+H	PE	1.66	0.03389	
PE(18:0p/18:1)+H	PE	1.60	0.03389	
PE(18:0p/18:2)+H	PE	1.57	0.03389	
PE(18:0p/20:3)+H	PE	0.94	0.03389	
PE(18:0p/20:4)+H	PE	1.36	0.03389	
PE(18:0p/21:1)+H	PE	1.20	0.03389	
PE(18:0p/22:3)+H	PE	1.26	0.03389	
PE(18:0p/22:4)+H	PE	1.32	0.03389	
PE(18:0p/22:5)+H	PE	1.57	0.03389	
PE(18:0p/22:6)+H	PE	1.44	0.03389	
PE(18:0p/24:1)+H	PE	1.56	0.03389	
PE(18:1/18:1)+H	PE	1.58	0.03389	
PE(18:1/20:4)+H	PE	1.41	0.03389	
PE(18:1/22:1)+H	PE	1.61	0.03389	
PE(18:1/22:4)+H	PE	1.42	0.03389	
PE(18:1/22:6)+H	PE	1.67	0.03389	
PE(18:1p/17:1)+H	PE	1.55	0.03389	
PE(18:1p/18:1)+H	PE	1.77	0.03389	

PE(18:1p/19:1)+H	PE	1.35	0.03389	
PE(18:1p/20:1)+H	PE	1.98	0.03389	
PE(18:1p/20:5)+H	PE	1.34	0.03389	
PE(18:1p/22:3)+H	PE	1.48	0.03389	
PE(18:1p/22:6)+H	PE	1.46	0.03389	
PE(18:1p/23:1)+H	PE	1.46	0.03389	
PE(18:1p/24:2)+H	PE	1.31	0.03389	
PE(18:2/20:4)+H	PE	1.73	0.03389	
PE(18:2/22:6)+H	PE	2.02	0.03389	
PE(18:2p/20:4)+H	PE	1.77	0.03389	
PE(18:2p/22:6)+H	PE	1.74	0.03389	
PE(20:0/22:6)+H	PE	1.26	0.03389	
PE(20:0p/22:6)+H	PE	1.43	0.03389	
PE(20:1/18:1)+H	PE	1.62	0.03389	
PE(20:1/22:6)+H	PE	1.18	0.03389	
PE(20:1p/22:6)+H	PE	1.31	0.03389	
PE(20:3/22:6)+H	PE	4.60	0.03389	
PE(20:4/22:6)+H	PE	1.54	0.03389	
PE(22:4/20:4)+H	PE	1.72	0.03389	
PE(22:4/22:6)+H	PE	1.61	0.03389	
PE(22:5/22:6)+H	PE	1.68	0.03389	
PE(22:6/22:6)+H	PE	1.58	0.03389	
PE(35:0)+H	PE	1.79	0.03389	
PE(38:4e)+H	PE	1.47	0.03389	
PE(38:4p)+H	PE	1.52	0.03389	
PE(38:5)+H	PE	1.50	0.03389	
PE(38:6e)+H	PE	1.61	0.03389	
PE(40:1)+H	PE	1.59	0.03389	
PE(40:5e)+H	PE	1.55	0.03389	
PE(40:7p)+H	PE	1.52	0.03389	
PE(42:4p)+H	PE	1.32	0.03389	
PE(44:2p)+H	PE	1.64	0.03389	

PEt(42:4)-H	PEt	0.94	0.03389	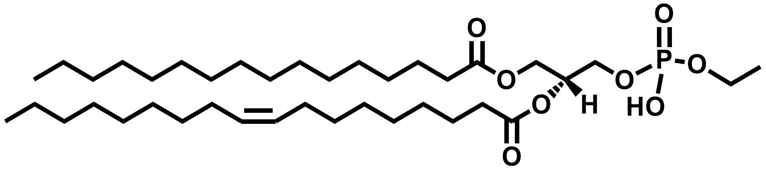 PEt (16:0/18:1)
PG(12:0/14:0)-H	PG	1.61	0.03389	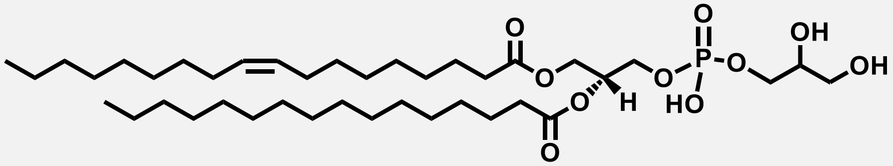 PG (18:1/16:0)
PG(16:0/16:0)-H	PG	3.49	0.03389	
PG(16:0/18:2)-H	PG	1.27	0.03389	
PG(17:0/17:0)-H	PG	1.48	0.03389	
PG(18:1/18:1)-H	PG	1.91	0.03389	
PI(16:0/22:4)-H	PI	1.70	0.03389	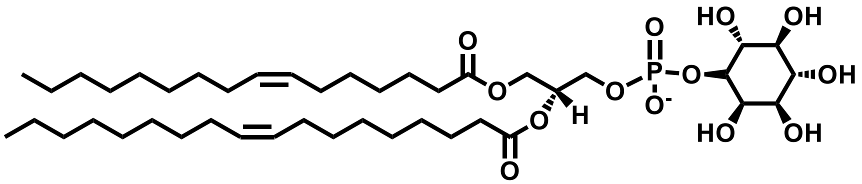 PI (16:1/18:0)
PI(16:1/18:1)-H	PI	1.55	0.03389	
PI(18:1/20:3)-H	PI	1.40	0.03389	
PI(18:1p/20:4)-H	PI	1.50	0.03389	
PI(35:0)-H	PI	1.74	0.03389	
PMe(16:0/16:1)-H	PMe	4.16	0.03389	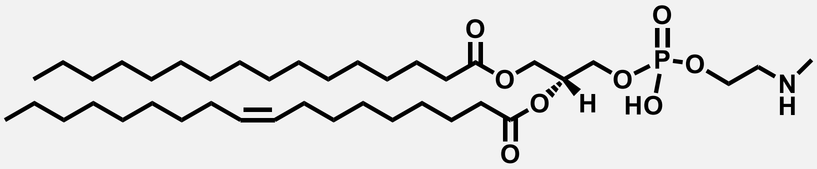 PMe (16:0/18:1)
PMe(16:0/18:1)-H	PMe	5.99	0.03389	
PMe(16:0/20:4)-H	PMe	6.80	0.03389	
PMe(18:0/18:1)-H	PMe	9.67	0.03389	
PMe(42:10)-H	PMe	5.87	0.03389	
PS(12:0/14:0)-H	PS	1.94	0.03389	
PS(18:1/22:0)-H	PS	1.00	0.03389	
PS(18:1/22:6)-H	PS	1.17	0.03389	
PS(20:0p/18:1)-H	PS	1.45	0.03389	
PS(20:1/18:1)-H	PS	1.36	0.03389	

PS(20:4/22:6)-H	PS	2.05	0.03389
PS(22:4/22:6)-H	PS	1.79	0.03389
PS(22:6/22:6)-H	PS	1.53	0.03389
PS(24:0/20:4)-H	PS	1.58	0.03389
PS(30:3)-H	PS	1.85	0.03389
PS(37:0)-H	PS	1.24	0.03389
PS(39:1)-H	PS	1.43	0.03389
PS(39:3)-H	PS	1.40	0.03389
PS(39:4)-H	PS	1.80	0.03389
PS(41:0)-H	PS	1.42	0.03389
PS(41:1)-H	PS	1.58	0.03389
PS(43:6)-H	PS	1.32	0.03389
PS(45:1)-H	PS	1.38	0.03389
SM(d18:0/18:1)+H	SM	1.39	0.03389
SM(d18:0/23:1)+H	SM	1.56	0.03389
SM(d18:1/14:0)+H	SM	1.73	0.03389
SM(d18:1/18:3)+H	SM	1.91	0.03389
SM(d18:1/20:1)+H	SM	1.61	0.03389
SM(d18:1/22:1)+H	SM	1.47	0.03389
SM(d18:1/23:1)+H	SM	1.42	0.03389
SM(d18:1/24:1)+H	SM	1.48	0.03389
SM(d18:1/24:2)+H	SM	1.81	0.03389
SM(d18:1/26:3)+H	SM	1.55	0.03389
SM(d18:1/28:1)+H	SM	1.96	0.03389
SM(d18:2/23:0)+H	SM	1.65	0.03389
SM(d33:1)+H	SM	1.91	0.03389
SM(d34:0)+H	SM	1.59	0.03389
SM(d34:1)+H	SM	1.56	0.03389
SM(d35:2)+H	SM	1.31	0.03389
SM(d36:0)+H	SM	1.58	0.03389
SM(d36:1)+H	SM	1.63	0.03389
SM(d38:1)+H	SM	1.75	0.03389
SM(d40:1)+H	SM	1.57	0.03389
SM(d40:2)+H	SM	3.01	0.03389

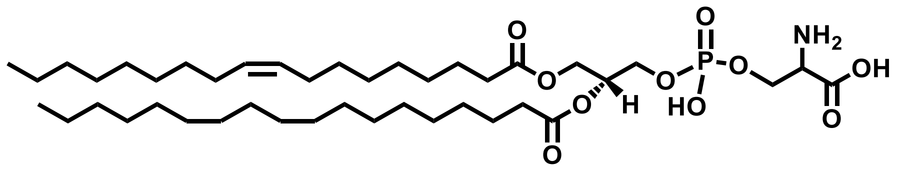

PS (18:1/18:2)

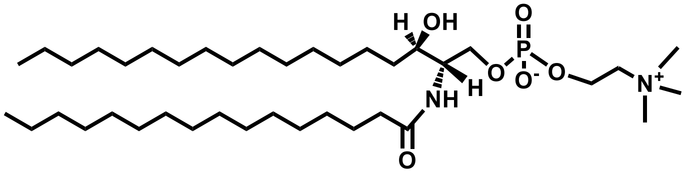

SM (d18:0/16:0)

SM(d44:3)+H	SM	1.80	0.03389	
So(d18:1)+H	So	1.56	0.03389	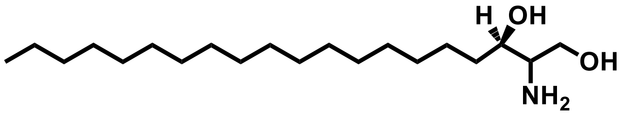 So (18:0)
TG(15:0/16:0/22:6)+NH4	TG	1.11	0.03389	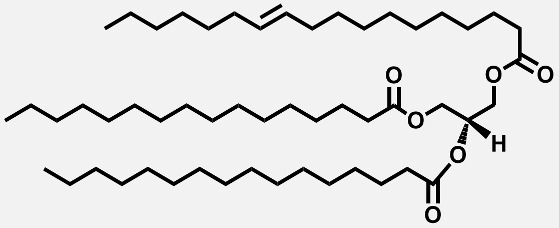 TG (16:0/16:0/18:1(11E))
TG(16:0/18:1/18:1)+NH4	TG	1.29	0.03389	
TG(16:0e/16:0/22:6)+NH4	TG	1.29	0.03389	
TG(18:0/18:0/20:1)+NH4	TG	1.14	0.03389	
TG(18:0/18:0/22:4)+NH4	TG	0.95	0.03389	
TG(18:0/18:1/20:0)+NH4	TG	1.53	0.03389	
TG(8:0/8:0/10:0)+NH4	TG	1.71	0.03389	
