# Supplementary material for: Influenza A virus infection disrupts oligodendrocyte homeostasis and alters the myelin lipidome in the adult mouse
Source: J Neuroinflammation. 2023 Aug 19;20:190. doi: 10.1186/s12974-023-02862-2 (PMC10439573; doi:10.1186/s12974-023-02862-2)
Supplement: Supplementary file 7 — Additional file 7: Table S6. List of all lipid species of purified mPFC myelin differentially expressed between flu-inoculated mice at day 8 and 16 p.i [file 12974_2023_2862_MOESM7_ESM.pdf]

Table S6. List of all lipid species of purified mPFC myelin differentially expressed between flu-inoculated mice at day 8 and 16 p.i.

mPFC (Flu day 8 vs. Flu day 16)				
Lipid Ion	***Lipid Class***	***Fold Change (Flu D16/Flu D8)***	***p-value***	***Representative Structures***
ChE(24:6)+NH4	ChE	0.02	0.03389	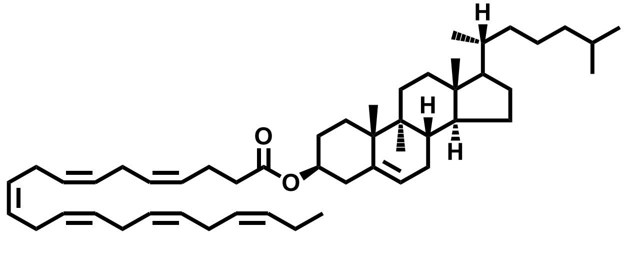 **ChE (22:6)**
CL(18:2/14:0/14:0/14:0)-H	CL	0.01	0.03389	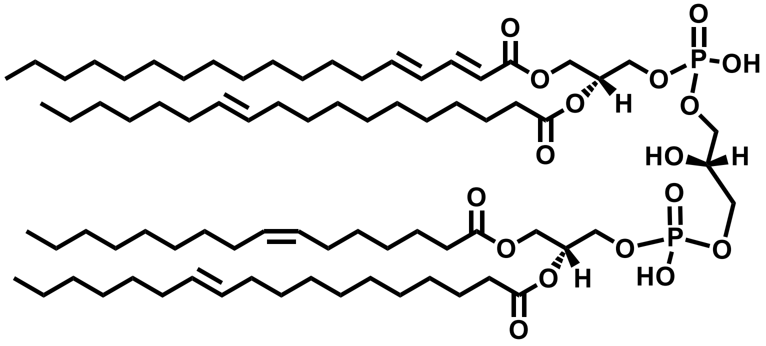 **CL (18:2/18:1/16:1/18:1)**
CL(20:4/18:1/16:1/18:1)-H	CL	1.01	0.03389	
dMePE(16:0/18:1)-H	dMePE	1.14	0.03389	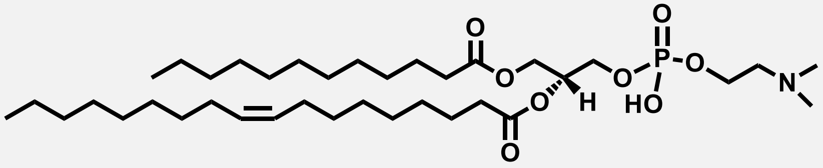 **dMePE (16:0/18:1)**

LPE(18:0p)-H	LPE	0.50	0.03389	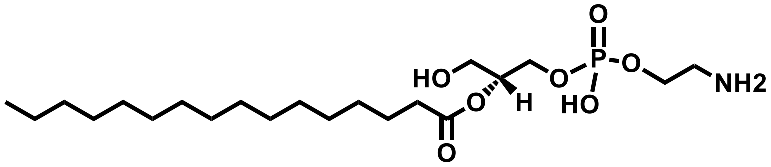<br>**LPE (16:0)**
MGDG(15:0/22:4)-H	MGDG	0.93	0.03389	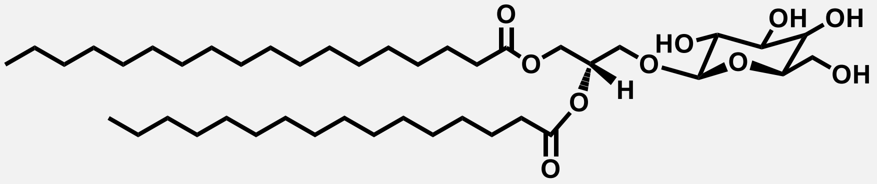<br>**MGDG (16:0/18:0)**
PE(17:0/18:1)+H	PE	1.50	0.03389	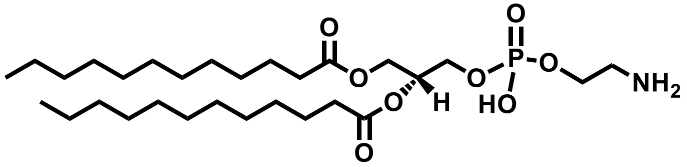<br>**PE (12:0/12:0)**
PE(18:1/18:3)+H	PE	1.08	0.03389	
PE(35:0)+H	PE	1.05	0.03389	
SM(d35:2)+H	SM	1.26	0.03389	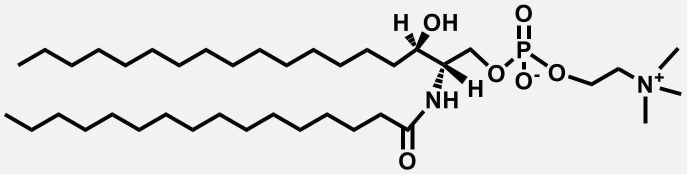<br>**SM (d18:0/16:0)**
SM(d37:1)+H	SM	1.13	0.03389	
SM(d40:7)+H	SM	1.24	0.03389	
So(d18:0)+H	So	1.05	0.03389	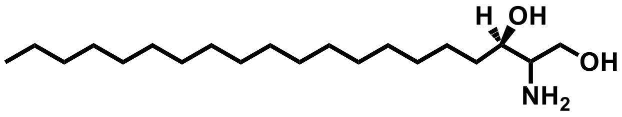<br>**So (18:0)**
TG(16:1/16:1/16:1)+NH4	TG	1.07	0.03389	
TG(16:1/16:1/18:2)+NH4	TG	1.11	0.03389	

TG(68:4)+NH4	TG	0.63	0.03389	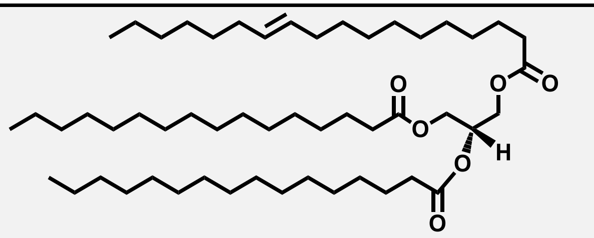 **TG (16:0/16:0/18:1(11E))**
