# Supplementary material for: Influenza A virus infection disrupts oligodendrocyte homeostasis and alters the myelin lipidome in the adult mouse
Source: J Neuroinflammation. 2023 Aug 19;20:190. doi: 10.1186/s12974-023-02862-2 (PMC10439573; doi:10.1186/s12974-023-02862-2)
Supplement: Supplementary file 8 — Additional file 8: Table S7. List of all lipid species of purified whole brain myelin differentially expressed between saline and flu-inoculated mice at day 8 p.i [file 12974_2023_2862_MOESM8_ESM.pdf]

Table S7. List of all lipid species of purified whole brain myelin differentially expressed between saline and flu-inoculated mice at day 8 p.i.

Myelin				
Lipid Ion	***Lipid Class***	***Fold Change (Flu/Saline)***	***p-value***	***Representative Structure***
AcCa(12:0)+H	*AcCa*	1.70	0.03301	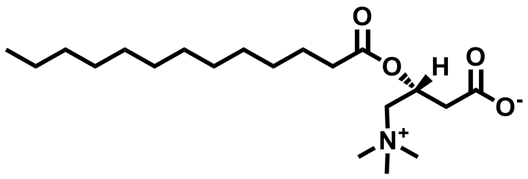 **AcCa (13:0)**
AcCa(13:0)+H	*AcCa*	2.56	0.00065	
AcCa(14:1)+H	*AcCa*	4.12	0.02759	
AcCa(20:5)+H	*AcCa*	1.78	0.04660	
AcCa(24:1)+H	*AcCa*	1.73	0.01052	
BisMePA(18:2p/16:1)+NH4	*BisMePA*	1.52	0.02759	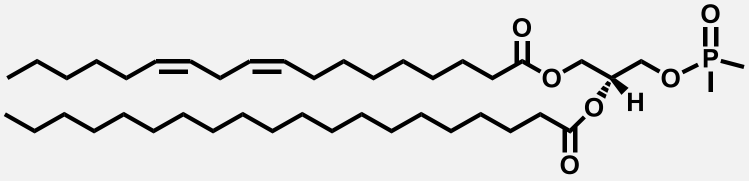 **BisMePA (18:2/20:0)**
BisMePA(16:0/20:4)+NH4	*BisMePA*	2.08	0.01568	
BisMePA(16:0/20:5)+NH4	*BisMePA*	2.61	0.03301	
BisMePA(18:2p/19:1)+NH4	*BisMePA*	2.55	0.04660	
BisMePA(18:2p/20:0)+NH4	*BisMePA*	2.02	0.03301	
BisMePA(18:0/20:5)+NH4	*BisMePA*	3.38	0.02759	
BisMePA(18:1/20:5)+NH4	*BisMePA*	2.60	0.01902	
BisMePA(18:0/22:5)+NH4	*BisMePA*	2.18	0.01287	
BisMePA(18:2p/22:3)+NH4	*BisMePA*	2.88	0.00855	
BisMePA(18:2p/22:5)+NH4	*BisMePA*	3.45	0.01902	
BisMePA(18:2p/22:6)+NH4	*BisMePA*	2.44	0.02296	
Cer(d18:0/18:0)+H	*Cer*	3.92	0.02296	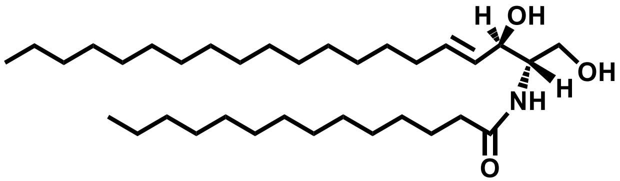 **Cer (18:1/14:0)**
Cer(d18:2/18:0)+H	*Cer*	5.68	0.01287	
Cer(d18:0/22:0)+H	*Cer*	1.89	0.03301	
Cer(d18:1/23:1)+H	*Cer*	2.45	0.01902	
Cer(d18:2/23:0)+H	*Cer*	2.44	0.02759	
Cer(d18:0/24:0)+H	*Cer*	1.43	0.03301	
Cer(d18:0/24:1)+H	*Cer*	2.46	0.01568	
Cer(d18:1/24:1)+H	*Cer*	3.05	0.04660	
Cer(d18:1/25:0)+H	*Cer*	1.45	0.04660	
Cer(d18:1/25:1)+H	*Cer*	1.73	0.01287	
Cer(d20:1/24:0)+H	*Cer*	1.93	0.01568	

Cer(d20:1/24:1)+H	*Cer*	1.72	0.01568
CerG1(d18:2/16:0)+H	*CerG1*	0.57	0.02759
CerG1(d18:1/18:0)+H	*CerG1*	2.39	0.03931
CerG1(d18:1/18:1)+H	*CerG1*	1.50	0.01287
CerG1(d18:1/20:0)+H	*CerG1*	2.04	0.02759
CerG1(d18:1/21:0)+H	*CerG1*	2.61	0.02759
CerG1(d18:1/21:1)+H	*CerG1*	2.04	0.01902
CerG1(d18:0/22:0)+H	*CerG1*	2.01	0.02759
CerG1(d18:2/22:1)+H	*CerG1*	2.18	0.03301
CerG1(d18:1/22:6)+H	*CerG1*	0.49	0.00692
CerG1(d18:0/23:1)+H	*CerG1*	3.73	0.00855
CerG1(d18:1/23:0)+H	*CerG1*	2.07	0.03301
CerG1(d18:2/23:0)+H	*CerG1*	3.34	0.01902
CerG1(d18:1/23:2)+H	*CerG1*	1.91	0.03301
CerG1(d18:2/23:1)+H	*CerG1*	1.94	0.01902
CerG1(d18:0/24:0)+H	*CerG1*	1.48	0.03301
CerG1(d18:1/24:0)+H	*CerG1*	1.93	0.04660
CerG1(d18:1/24:1)+H	*CerG1*	3.07	0.03931
CerG1(d18:2/24:1)+H	*CerG1*	1.99	0.03301
CerG1(d18:1/24:2)+H	*CerG1*	3.16	0.03931
CerG1(d18:2/24:2)+H	*CerG1*	1.58	0.02759
CerG1(d18:1/24:3)+H	*CerG1*	2.83	0.02759
CerG1(d18:0/25:1)+H	*CerG1*	2.28	0.01287
CerG1(d18:1/25:1)+H	*CerG1*	1.92	0.03931
CerG1(d18:2/25:1)+H	*CerG1*	1.96	0.01287
CerG1(d18:1/25:2)+H	*CerG1*	1.79	0.02759
CerG1(d18:1/26:1)+H	*CerG1*	2.60	0.03931
CerG1(d18:1/26:2)+H	*CerG1*	9.37	0.02759
CerG1(d44:4)+H	*CerG1*	19.23	0.03301
CerG2(d34:2)+H	*CerG2*	3.53	0.01568
CerG2(d18:1/22:6)+H	*CerG2*	0.50	0.03931
CerG2(d18:1/24:0)+H	*CerG2*	2.55	0.01902
CerG2(d54:4)+H	*CerG2*	1.90	0.01287

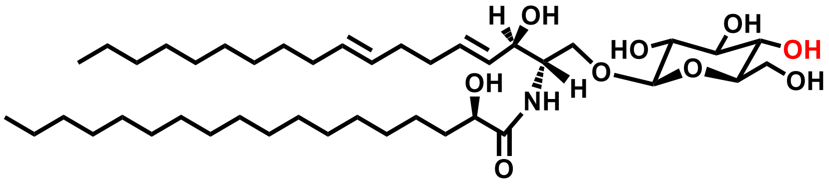

Glucosylceramide (18:2/18:0)

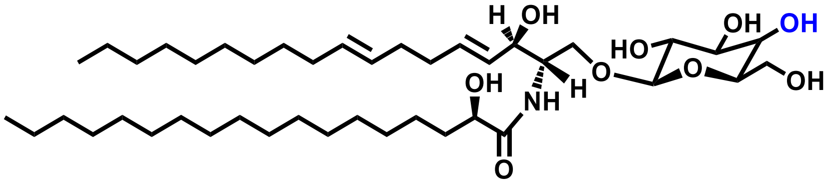

Galactosylceramide (18:2/18:0)

ChE(22:6)+NH4	*ChE*	1.83	0.02759	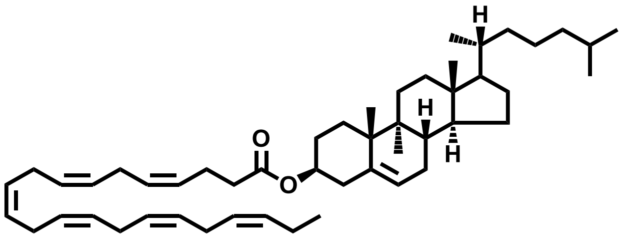 **ChE (22:6)**
ChE(24:6)+NH4	*ChE*	1.64	0.03301	
CL(18:2/18:1/16:1/18:1)-H	*CL*	1.32	0.02759	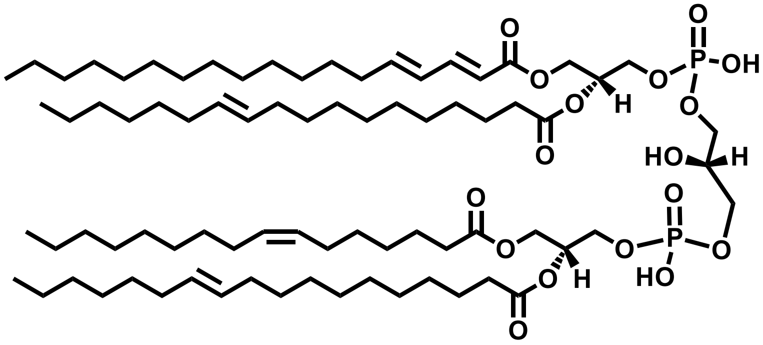 **CL (18:2/18:1/16:1/18:1)**
CL(20:4/18:1/16:1/18:1)-H	*CL*	1.21	0.02759	
CL(18:2/18:1/20:4/18:1)-H	*CL*	1.57	0.01287	
CL(17:1/18:1/20:4/22:6)-H	*CL*	2.11	0.03931	
CL(23:0/20:4/18:0/20:4)-H	*CL*	2.28	0.03019	
DG(16:0/16:0)+NH4	*DG*	3.31	0.03931	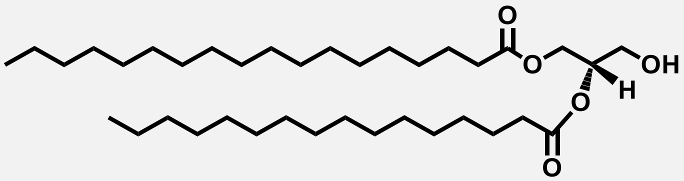 **DG (16:0/18:0)**
DG(16:0/16:1)+NH4	*DG*	3.34	0.00855	
DG(18:0/16:0)+NH4	*DG*	2.55	0.02296	
DG(18:0/18:2)+NH4	*DG*	3.24	0.03931	
DG(18:1/18:2)+NH4	*DG*	5.12	0.00050	
DG(16:1/20:4)+NH4	*DG*	2.76	0.03931	
DG(20:1/18:2)+NH4	*DG*	2.05	0.03931	
DG(18:0/20:4)+NH4	*DG*	2.23	0.02296	
DG(22:1/18:2)+NH4	*DG*	2.12	0.01568	
DG(18:0/22:3)+NH4	*DG*	1.86	0.03931	
DG(18:0/22:6)+NH4	*DG*	2.75	0.03301	
DG(18:2/23:0)+NH4	*DG*	1.47	0.01052	
DG(18:1/24:2)+NH4	*DG*	1.58	0.01568	
DG(20:4/23:0)+NH4	*DG*	1.38	0.02759	
DG(24:1/20:4)+NH4	*DG*	1.60	0.01287	

LPA(18:0)-H	*LPA*	0.50	0.01902	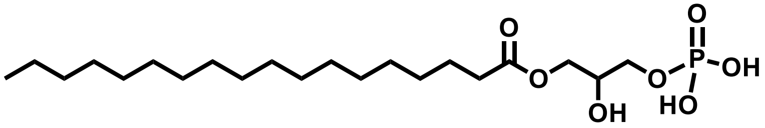 = **LPA (18:0)**
LPC(26:0)+H	*LPC*	2.09	0.03301	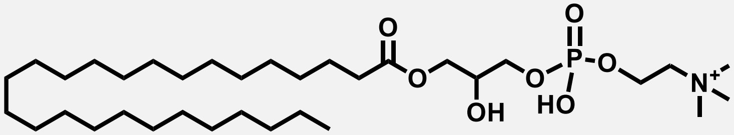 **LPC (26:0)**
LPS(16:0)-H	*LPS*	0.40	0.01052	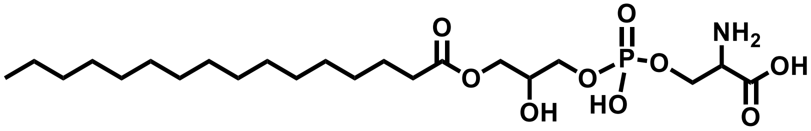 **LPS (16:0)**
LPS(18:1)-H	*LPS*	0.58	0.01287	
LPS(20:4)-H	*LPS*	0.35	0.01902	
MePC(32:2)+NH4	*MePC*	2.07	0.01287	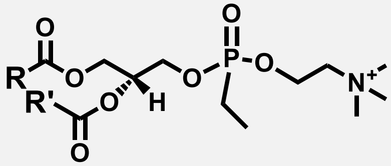 **MePC**
MePC(34:4)+NH4	*MePC*	2.61	0.04660	
MePC(34:5)+NH4	*MePC*	1.77	0.01568	
MePC(42:5)+NH4	*MePC*	4.36	0.01902	
MG(32:0)+H	*MG*	2.02	0.02296	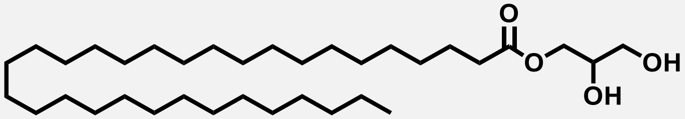 **MG (32:0)**
MG(36:0)+H	*MG*	1.57	0.02759	
PC(23:3e)+H	*PC*	0.63	0.04660	
PC(24:4e)+H	*PC*	0.66	0.03301	
PC(31:0)+H	*PC*	2.71	0.04660	
PC(31:3)+H	*PC*	1.09	0.04660	
PC(33:0p)+H	*PC*	2.27	0.04660	

PC(17:1/16:1)+H	*PC*	2.09	0.03931	
PC(33:4)+H	*PC*	1.97	0.02759	
PC(18:0p/16:1)+H	*PC*	2.04	0.03931	
PC(34:2)+H	*PC*	2.17	0.02296	
PC(35:5)+H	*PC*	1.90	0.03301	
PC(18:1/18:1)+H	*PC*	3.20	0.03301	
PC(36:2e)+H	*PC*	1.97	0.03931	
PC(37:0)+H	*PC*	1.62	0.04660	
PC(37:5)+H	*PC*	1.16	0.04660	
PC(38:4)+H	*PC*	4.05	0.03301	
PC(18:3/20:5)+H	*PC*	1.97	0.04660	
PC(40:3p)+H	*PC*	1.80	0.04660	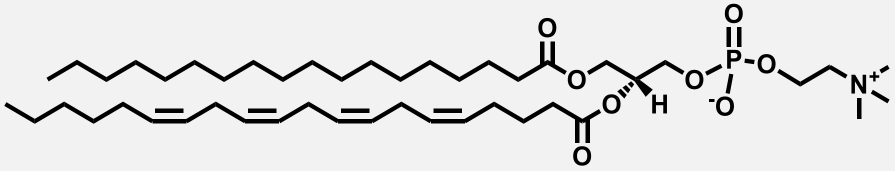
PC(18:0p/22:6)+H	*PC*	1.85	0.03931	
PC(40:6p)+H	*PC*	5.24	0.03931	
PC(18:1/22:6)+H	*PC*	2.73	0.04660	**PC (18:0/20:4)**
PC(18:2/23:1)+H	*PC*	1.92	0.01902	
PC(42:11)+H	*PC*	2.87	0.00855	
PC(42:3)+H	*PC*	2.42	0.03931	
PC(42:5)+H	*PC*	2.52	0.02759	
PC(43:3)+H	*PC*	1.95	0.01568	
PC(43:4)+H	*PC*	1.70	0.02759	
PC(44:5)+H	*PC*	2.96	0.02759	
PC(45:4)+H	*PC*	2.29	0.01902	
PC(45:5)+H	*PC*	1.69	0.03301	
PC(46:5)+H	*PC*	1.77	0.03931	
PC(60:6)+H	*PC*	2.72	0.01902	
PE(20:3e)+H	*PE*	1.65	0.03931	
PE(16:0/16:1)+H	*PE*	2.62	0.01052	
PE(18:1p/16:1)+H	*PE*	1.95	0.01902	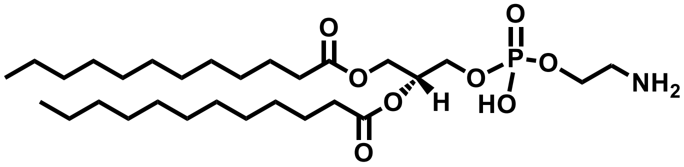
PE(16:1/18:2)+H	*PE*	2.10	0.01287	
PE(16:0p/20:0)+H	*PE*	2.11	0.04660	
PE(18:0/18:1)+H	*PE*	2.39	0.03931	**PE (12:0/12:0)**
PE(16:0/20:4)+H	*PE*	2.61	0.03301	
PE(18:0p/19:1)+H	*PE*	2.06	0.04660	

PE(18:1p/19:1)+H	*PE*	2.42	0.03931	
PE(18:0p/20:1)+H	*PE*	1.95	0.04660	
PE(18:0p/20:3)+H	*PE*	2.14	0.04660	
PE(18:0/20:4)+H	*PE*	3.34	0.03301	
PE(38:4e)+H	*PE*	8.39	0.01568	
PE(18:1/20:4)+H	*PE*	2.59	0.02296	
PE(18:2/20:4)+H	*PE*	1.18	0.01902	
PE(16:0/22:6)+H	*PE*	2.29	0.04660	
PE(18:2p/20:4)+H	*PE*	2.73	0.00224	
PE(16:0p/22:6)+H	*PE*	2.71	0.03931	
PE(16:1p/22:6)+H	*PE*	1.30	0.03301	
PE(40:3p)+H	*PE*	4.64	0.02759	
PE(18:0p/22:4)+H	*PE*	2.16	0.03931	
PE(18:0/22:6)+H	*PE*	2.83	0.03931	
PE(18:1p/22:5)+H	*PE*	2.12	0.03301	
PE(18:0p/22:6)+H	*PE*	3.36	0.01902	
PE(18:1/22:6)+H	*PE*	2.79	0.02759	
PE(18:2/22:6)+H	*PE*	1.43	0.03301	
PE(18:1p/24:2)+H	*PE*	1.61	0.02759	
PE(20:0p/22:4)+H	*PE*	2.25	0.03931	
PE(42:4p)+H	*PE*	7.19	0.01568	
PE(24:0/20:4)+H	*PE*	2.34	0.01568	
PE(44:4)+H	*PE*	4.21	0.01287	
PEt(16:0/14:0)-H	*PEt*	0.43	0.03301	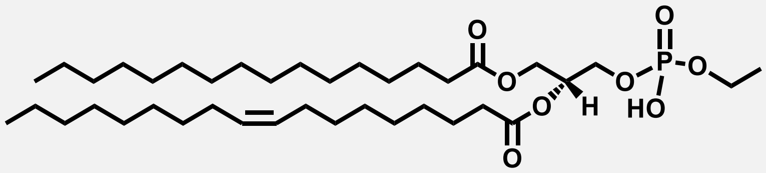 **PEt (16:0/18:1)**

PG(18:0/16:0)-H	*PG*	1.43	0.03931	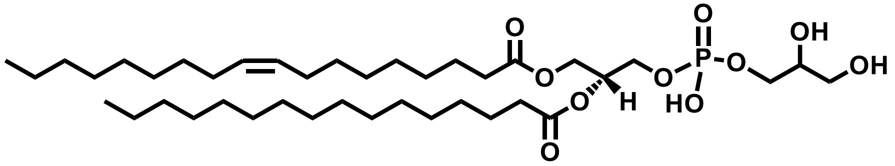 **PG (18:1/16:0)**
PS(18:1/18:2)-H	*PS*	1.84	0.03301	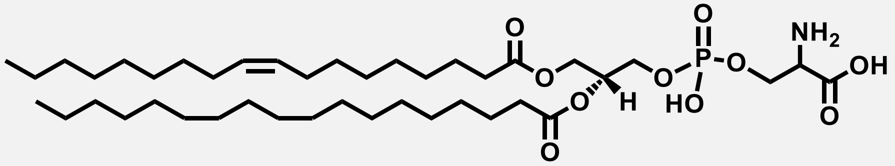 **PS (18:1/18:2)**
SM(d35:1)+H	*SM*	2.09	0.02759	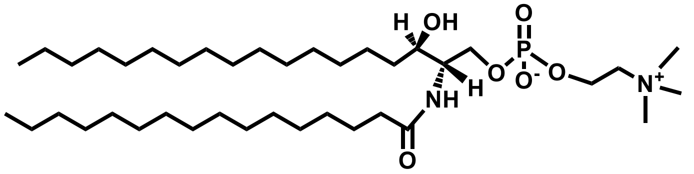 **SM (d18:0/16:0)**
SM(d36:0)+H	*SM*	3.48	0.03301	
SM(d18:0/18:1)+H	*SM*	4.03	0.03301	
SM(d18:1/18:1)+H	*SM*	2.58	0.01568	
SM(d18:2/18:1)+H	*SM*	0.61	0.04660	
SM(d18:1/20:1)+H	*SM*	2.82	0.03301	
SM(d18:1/22:0)+H	*SM*	2.49	0.03301	
SM(d18:1/23:0)+H	*SM*	2.01	0.01287	
SM(d18:1/25:1)+H	*SM*	1.78	0.01902	
SM(d18:1/25:2)+H	*SM*	2.24	0.03931	
SM(d18:1/26:0)+H	*SM*	1.34	0.04660	
SM(d18:1/26:1)+H	*SM*	1.84	0.03301	
SM(d18:1/26:4)+H	*SM*	96.47	0.01287	
TG(40:4e)+NH4	*TG*	1.93	0.04660	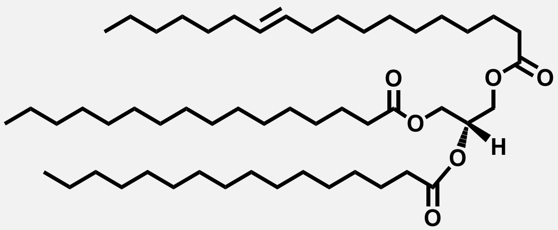 **TG (16:0/16:0/18:1(11E))**
TG(42:3e)+NH4	*TG*	1.63	0.01287	
TG(43:4e)+NH4	*TG*	1.38	0.02759	
TG(14:0e/10:1/20:4)+NH4	*TG*	1.60	0.01287	
TG(16:0/16:0/17:0)+NH4	*TG*	3.17	0.03931	
